# Mutation in the intracellular chloride channel CLCC1 associated with autosomal recessive retinitis pigmentosa

**DOI:** 10.1371/journal.pgen.1007504

**Published:** 2018-08-29

**Authors:** Lin Li, Xiaodong Jiao, Ilaria D’Atri, Fumihito Ono, Ralph Nelson, Chi-Chao Chan, Naoki Nakaya, Zhiwei Ma, Yan Ma, Xiaoying Cai, Longhua Zhang, Siying Lin, Abdul Hameed, Barry A. Chioza, Holly Hardy, Gavin Arno, Sarah Hull, Muhammad Imran Khan, James Fasham, Gaurav V. Harlalka, Michel Michaelides, Anthony T. Moore, Zeynep Hande Coban Akdemir, Shalini Jhangiani, James R. Lupski, Frans P. M. Cremers, Raheel Qamar, Ahmed Salman, John Chilton, Jay Self, Radha Ayyagari, Firoz Kabir, Muhammad Asif Naeem, Muhammad Ali, Javed Akram, Paul A. Sieving, Sheikh Riazuddin, Emma L. Baple, S. Amer Riazuddin, Andrew H. Crosby, J. Fielding Hejtmancik

**Affiliations:** 1 Department of Ophthalmology, Shanghai Ninth People’s Hospital, Shanghai JiaoTong University School of Medicine, Shanghai, P.R. China; 2 Ophthalmic Genetics and Visual Function Branch, National Eye Institute, National Institutes of Health, Bethesda, Maryland, United States of America; 3 RILD Wellcome Wolfson Centre, Royal Devon & Exeter NHS Foundation Trust, Exeter, United Kingdom; 4 Section on Model Synaptic Systems, Laboratory of Molecular Physiology, National Institute on Alcohol Abuse and Alcoholism, National Institutes of Health, Bethesda, Maryland, United States of America; 5 Department of Physiology, Osaka Medical College, Takatsuki, Japan; 6 Unit on Neural Circuits, National Institute of Neurological Disorders and Stroke, National Institutes of Health, Bethesda, Maryland, United States of America; 7 Laboratory of Immunology, National Eye Institute, National Institutes of Health, Bethesda, Maryland, United States of America; 8 Section of Molecular Mechanisms of Glaucoma, Laboratory of Molecular and Developmental Biology, National Eye Institute, National Institutes of Health, Bethesda, Maryland, United States of America; 9 School of Life Sciences, University of Science and Technology of China, Hefei, Anhui, P.R. China; 10 Institute of Biomedical and Genetic Engineering (IBGE), Islamabad, Pakistan; 11 Institute of Ophthalmology, University College London, London, United Kingdom; 12 Department of Biosciences, Moorfields Eye Hospital, London, United Kingdom; 13 Faculty of Science, COMSATS Institute of Information Technology, Islamabad, Pakistan; 14 Department of Clinical Genetics, Royal Devon & Exeter NHS Foundation Trust, Exeter, United Kingdom; 15 Ophthalmology Department, UCSF School of Medicine, San Francisco, California, United States of America; 16 Department of Molecular and Human Genetics, Baylor College of Medicine, Houston, Texas, United States of America; 17 Human Genome Sequencing Center, Baylor College of Medicine, Houston, Texas, United States of America; 18 Department of Pediatrics, Baylor College of Medicine, Houston, Texas, United States of America; 19 Texas Children’s Hospital, Houston, Texas, United States of America; 20 Department of Human Genetics, Donders Institute for Brain, Cognition and Behaviour, Radboud University Medical Center, Nijmegen, The Netherlands; 21 Faculty of Medicine, University of Southampton, Southampton, United Kingdom; 22 Shiley Eye Institute, University of California San Diego, La Jolla, California, United States of America; 23 National Centre of Excellence in Molecular Biology, University of the Punjab, Lahore, Pakistan; 24 The Wilmer Eye Institute, Johns Hopkins University School of Medicine, Baltimore, Maryland, United States of America; 25 Allama Iqbal Medical College, University of Health Sciences, Lahore, Pakistan; 26 National Centre for Genetic Diseases, Shaheed Zulfiqar Ali Bhutto Medical University, Islamabad, Pakistan; 27 National Eye Institute, National Institutes of Health, Bethesda, Maryland, United States of America; Max Planck Institute for Molecular Genetics, GERMANY

## Abstract

We identified a homozygous missense alteration (c.75C>A, p.D25E) in *CLCC1*, encoding a presumptive intracellular chloride channel highly expressed in the retina, associated with autosomal recessive retinitis pigmentosa (arRP) in eight consanguineous families of Pakistani descent. The p.D25E alteration decreased CLCC1 channel function accompanied by accumulation of mutant protein in granules within the ER lumen, while siRNA knockdown of *CLCC1* mRNA induced apoptosis in cultured ARPE-19 cells. TALEN KO in zebrafish was lethal 11 days post fertilization. The depressed electroretinogram (ERG) cone response and cone spectral sensitivity of 5 dpf KO zebrafish and reduced eye size, retinal thickness, and expression of rod and cone opsins could be rescued by injection of wild type *CLCC1* mRNA. *Clcc1*^*+/-*^ KO mice showed decreased ERGs and photoreceptor number. Together these results strongly suggest that intracellular chloride transport by CLCC1 is a critical process in maintaining retinal integrity, and CLCC1 is crucial for survival and function of retinal cells.

## Author summary

Retinitis pigmentosa (RP) is a group of conditions that affect the functioning of the light receptor cells in the eye. The first symptom of RP is usually reduced night vision, then loss of peripheral vision, then later loss of central vision. It can eventually lead to complete blindness. A number of genes have previously been shown to cause RP. In this paper we present eight families, all likely to share a common Pakistani ethnicity, each with multiple members with autosomal recessive RP. They all share a newly-identified homozygous genetic variant in the gene *CLCC1*, encoding a putative protein channel present inside all cells. Our studies demonstrate that alterations to this gene and protein channel specifically affect cell survival and eye development in cell culture, as well as retinal structure and physiology in genetically-altered zebrafish and mice. This is the first identification of *CLCC1* as a cause of human disease and enables improved understanding of the molecules important for eye development and function, as well as providing a genetic diagnosis for families with RP. Subsequent studies of this molecule may ultimately suggest novel treatment approaches.

## Introduction

Retinitis pigmentosa (RP [MIM 268000]) comprises a group of phenotypically and genetically heterogeneous inherited retinal diseases. RP is estimated to have a worldwide prevalence of approximately 1 in 4,000 [1]. The initial symptom of RP is usually night blindness, followed by loss of peripheral vision and, later in the disease, loss of central vision. In some cases this leads to complete blindness, although central visual acuity can be preserved until late in the disease. Rod photoreceptors are affected initially with subsequent cone photoreceptor degeneration as the disease progresses [2]. Affected individuals often have decreased or even undetectable rod responses in their electroretinogram (ERG) recordings even in early stages of the disease. The classic presenting symptoms of RP, problems with dark adaptation and night blindness, occur early in arRP (median age of onset 11 years) and later in autosomal dominant RP (adRP, median age of onset 24 years).[2] Despite this, a clinical diagnosis of RP may be delayed [3].

From a large series, dominant disease accounted for 20% of cases, recessive (classified based on consanguinity or >1 affected sibling) for 15%, X-linked for 7%, with 43% sporadic/simplex cases [4]. Sporadic cases are often assumed to be recessive in origin although a significant minority will represent other genetic causes including *de novo* dominant disease, X-linked (for males), mitochondrial or uniparental isodisomy [1]. To date, more than 22 genes causing adRP and 39 genes causing arRP are listed in the RetNet database, although, depending on the population being studied, these genes account for less than 40–60% of all cases of RP [5]. It is likely that the remaining cases have mutations in genes that have not yet been identified or involve intronic or regulatory regions of known genes. Many of the genes implicated in the RP disease process can be categorized by function or pathway, providing insight into retinal biology and the disease process. Important among these gene classes are those which code for: components of the phototransduction cascade, enzymes involved in retinol metabolism, cell surface proteins that act in cell-cell interactions, photoreceptor structural proteins, transcription factors, intracellular transport proteins, and splicing factors [1].

We previously mapped a new arRP locus (RP32) in a consanguineous Pakistani family with clinical findings typical of arRP (Family 1, [61030]), to a 10.5cM (8.9Mb) interval on chromosome 1p13.3–p21.2 [6]. This interval, flanked by markers D1S2896 and D1S457, was near but distinct from *ABCA4* [7,8], as shown by obligate recombinants at 7 microsatellite markers including D1S2896. In this study we discovered, within the refined genetic mapping interval, a c.75C>A (p.D25E) alteration in *CLCC1*, encoding a putative intracellular chloride channel expressed at high levels in the retina in this and 7 additional Pakistani-derived families with arRP. Together with supportive functional studies presented here which include channel activity, cell localization, and zebrafish and mouse model investigations, our data defines CLCC1 as a molecule that plays a critical role in retinal cell survival, development, and maintenance, and determines that its absence causes retinal degeneration.

## Results

### Genetic studies

**Fig 1:**
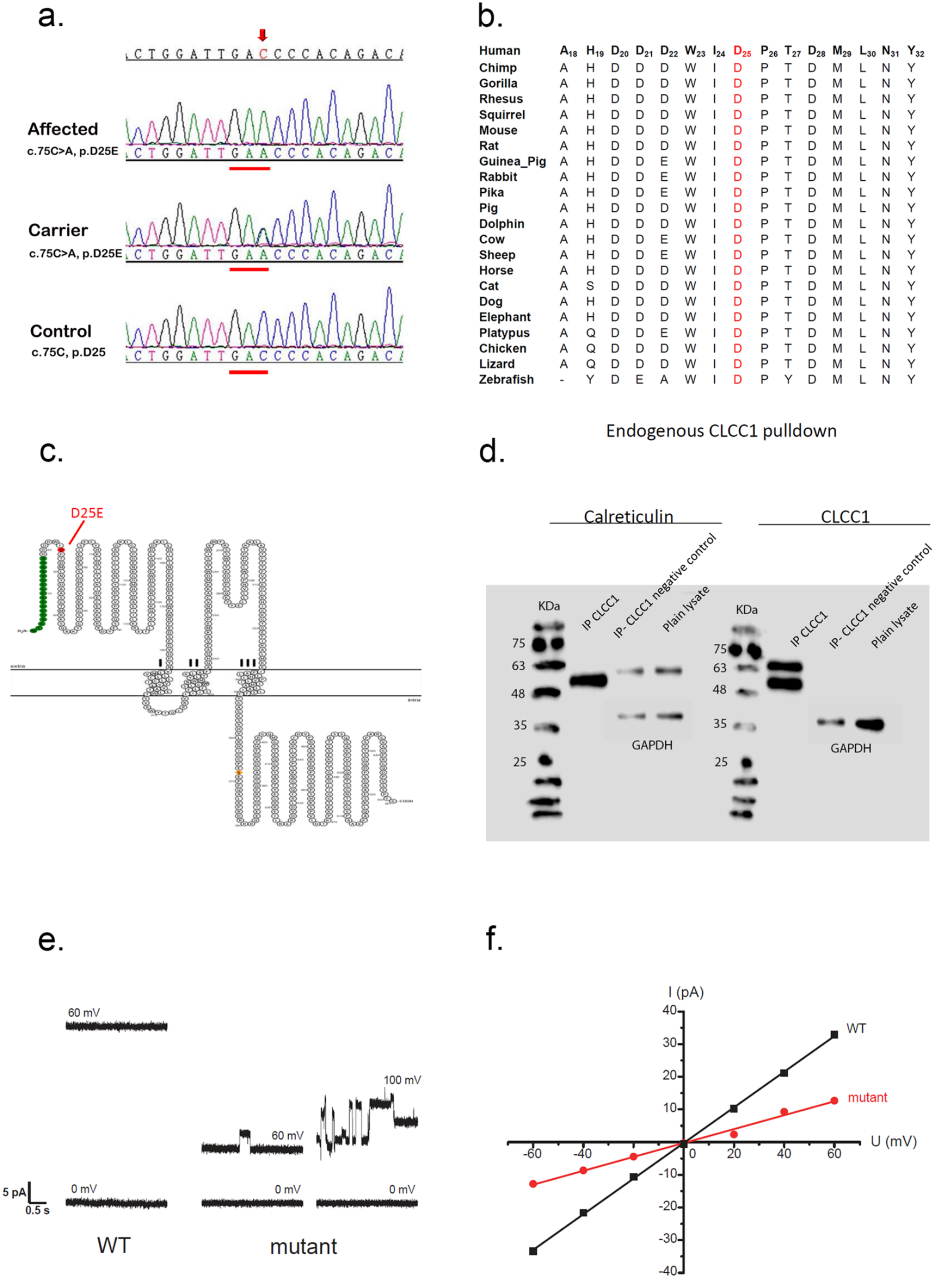
CLCC1 region, sequence, protein domains, and function. (a) Electropherograms: the sequence of an affected person (top, Individual 27, Family 2), the sequence of a heterozygous carrier (middle, Individual 29, Family 2) and unaffected control sequence (bottom) surrounding the *CLCC1* c.75C>A alteration, electropherograms of additional families are similar, but are not shown. (b) Amino acid sequence alignment around the D25 amino acid of CLCC1 (red) in 22 species ranging from human to zebrafish. D25 is absolutely conserved in all species and the entire region is relatively well conserved, especially among mammals. (c) Analysis with the online PSORT algorithm (https://wolfpsort.hgc.jp/) predicts the presence of a signal peptide at the N-terminal of CLCC1 and three transmembrane domains. (d) Co-immunoprecipitation demonstrates interaction of CLCC1 with Calreticulin. (e) Representative current traces of microsomes from WT and mutant cells. The recording voltage was marked above each trace. The mutant shows clear open/close channel activities while the WT does not in this tracing. The same buffer (5 mM Tris, 5 mM MOPS, 150 mM KCl, pH 7.0) was used on both sides. (f) The current-voltage (I/V) plot of microsomes from WT and mutant cells demonstrating decreased channel activity of the p.D25E mutant relative to the WT.

We investigated eight families of Pakistani descent with multiple individuals affected by arRP of as part of two independent, parallel research studies. Microsatellite fine mapping in a the previously presented Family 1 identified the chromosome 1p13.3 arRP locus and further confirmed homozygosity across the critical interval, but was unable to refine it further (S1 Table). To identify the causative gene, a combination of dideoxy and whole exome sequencing was undertaken in a single affected family member (Individual 12, Family 1), which identified only two non-synonymous homozygous variants in the linked region (S1 Table). The first of these, rs750180668, a homozygous c.75C>A, p.D25E missense mutation in exon 1 of the putative chloride channel gene CLIC-like 1 (*CLCC1*), was confirmed by dideoxy sequencing in all affected individuals (Fig 1a). In contrast, a second variation in a nearby gene *C1orf194* did not cosegregate with the arRP phenotype, evidenced by Individual 17, Family 1 who is heterozygous for the *C1orf194* variant, but affected with arRP (S1 Fig). Dideoxy sequencing of *CLCC1* in 123 additional consanguineous Pakistani arRP families revealed 4 additional apparently unrelated families in which the same c.75C>A, p.D25E alteration cosegregated with arRP (Families 2 [61031], 3 [61328], 4 [61244], and 5 [61224] S1 Table). All five families were found to be linked to markers in the 3.4cM (3.05 Mb) region and to the *CLCC1* c.75C>A variant, producing maximum LOD scores of 8.9, 5.0, 3.86, 2.44, and 2.45 at θ = 0 (with the *CLCC1* variant), respectively (S1 Table). All affected individuals share a common *CLCC1* intragenic SNP haplotype, indicative of a founder mutation with p<10^−8^ (S2 Table).

The above studies are entirely complementary to investigations of two previously unpublished families from Pakistan (Families 6 and 7), as well as a British-Bangladeshi family (Family 8; Moorfields Eye Hospital case GC17026) (S1 Fig), undertaken in parallel and independently of the above investigations. Whole genome SNP mapping of these families identified a 1.2Mb region of homozygosity of chromosome 1p13.3 common to all affected family members in all three families (flanked by markers rs17020437 and rs587727), indicative of a single ancestral founder mutation as the cause of the condition. When all eight families were subjected to SNP mapping, the autozygous region common to all affected family members was further refined to a small 322kb interval flanked by rs1333130-rs587727 (chr1: 108,820,610-109,142,651, hg38, S2 Fig), notably refining the extent of the original critical region and further confirming exclusion of the *C1orf194* variant. Whole exome sequencing of individuals from families 6, 7 and 8 (Individual 5, Family 6; Individual 8, Family 7; Individual 3, Family 8) identified only a single deleterious sequence variant in the critical region, the same p.D25E (c.75C>A) CLCC1 amino acid substitution. Sharing of the identical SNP haplotype (S2 Fig), which is estimated to have a frequency of 0.03 by the EM algorithm as incorporated into the Golden Helix SVS program (Bozeman MT), indicates that arRP in all eight families arises due to homozygosity for the same ancestral mutation (p = 2x10^-11^). This includes the British-Bangladeshi family (Family 8), which is also likely to be of the same Pakistani (Punjab) origin.

**Fig 2:**
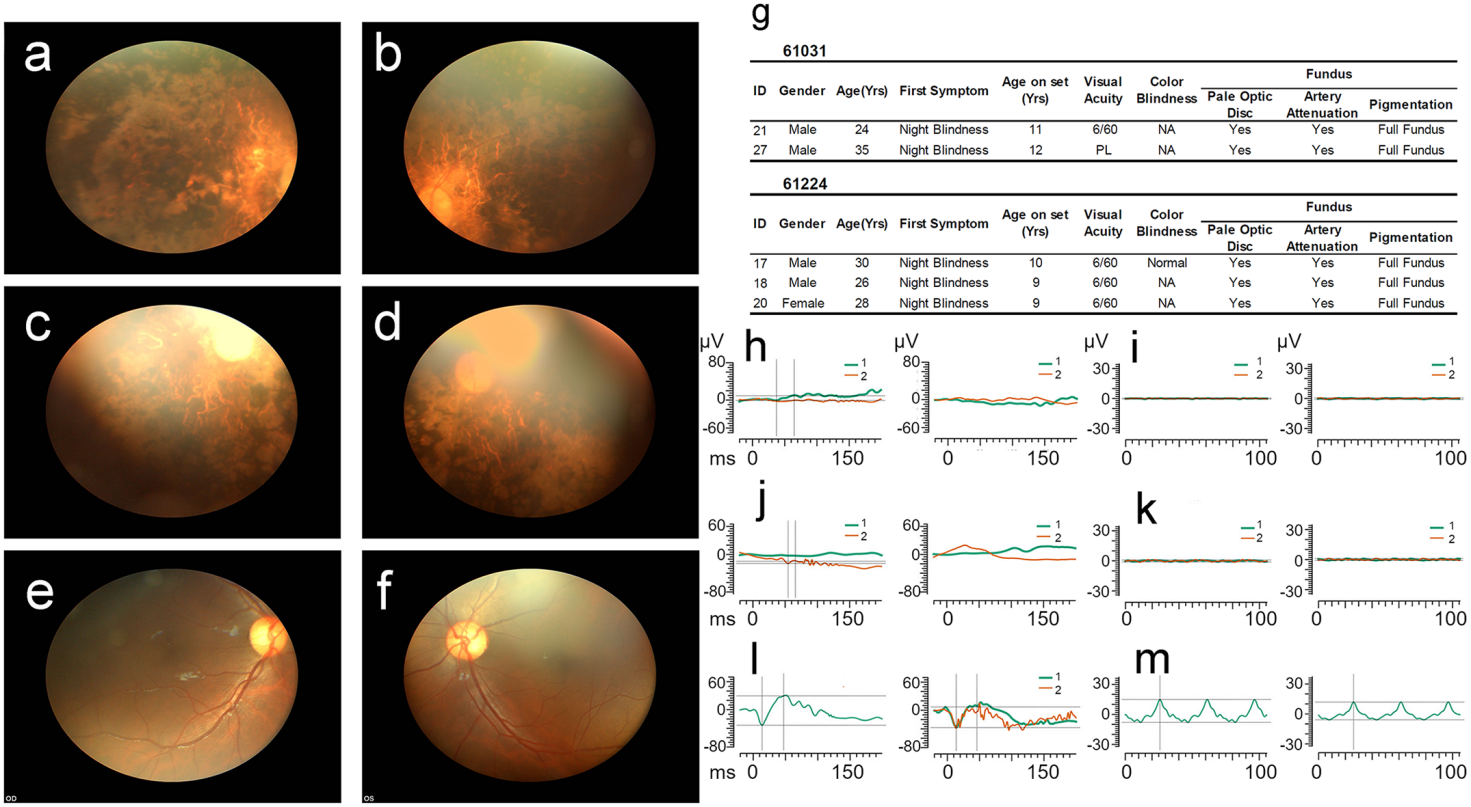
Fundus photographs and clinical findings. Fundus photographs of Individual 21 of Family 2 (a and b: OD [right eye] and OS [left eye], respectively), Individual 17 of Family 5 (c and d: OD and OS, respectively) and Individual 20 of Family 6 (n and o: OD and OS respectively) revealed findings similar to those seen in Family 1, including obvious bone spicule-shaped pigment deposits in the mid-periphery, waxy-pale optic discs, attenuation of the retinal arteries, and a generalized grayish carpet-like retinal degeneration as compared to a normal fundus (e and f: OD and OS, respectively). Maculopathy was detected in Family 1 and Family 6 but was not observed in Families 2 and 5. (g) Clinical findings of Family 2 and 5; the presenting symptom in both families is reported to be night blindness by approximately 10 years of age. All affected family members have moderate loss of visual acuity. The clinical characteristics of Family 1 were previously published [6]. h) rod (green) and cone (red) response: OD & OS, respectively; and i) 30Hz flicker response: OD & OS, respectively of Individual 21; Family 2. j) rod (green) and cone (red) response: OD & OS, respectively; and k) 30Hz flicker response of Individual 17 of Family 5. l) rod (green) and cone (red) response: OD & OS, respectively; and m) 30Hz flicker response of an age- and ethnically-matched control. The affected individuals demonstrate loss of ERG responses in keeping with advanced RP.

**Fig 3:**
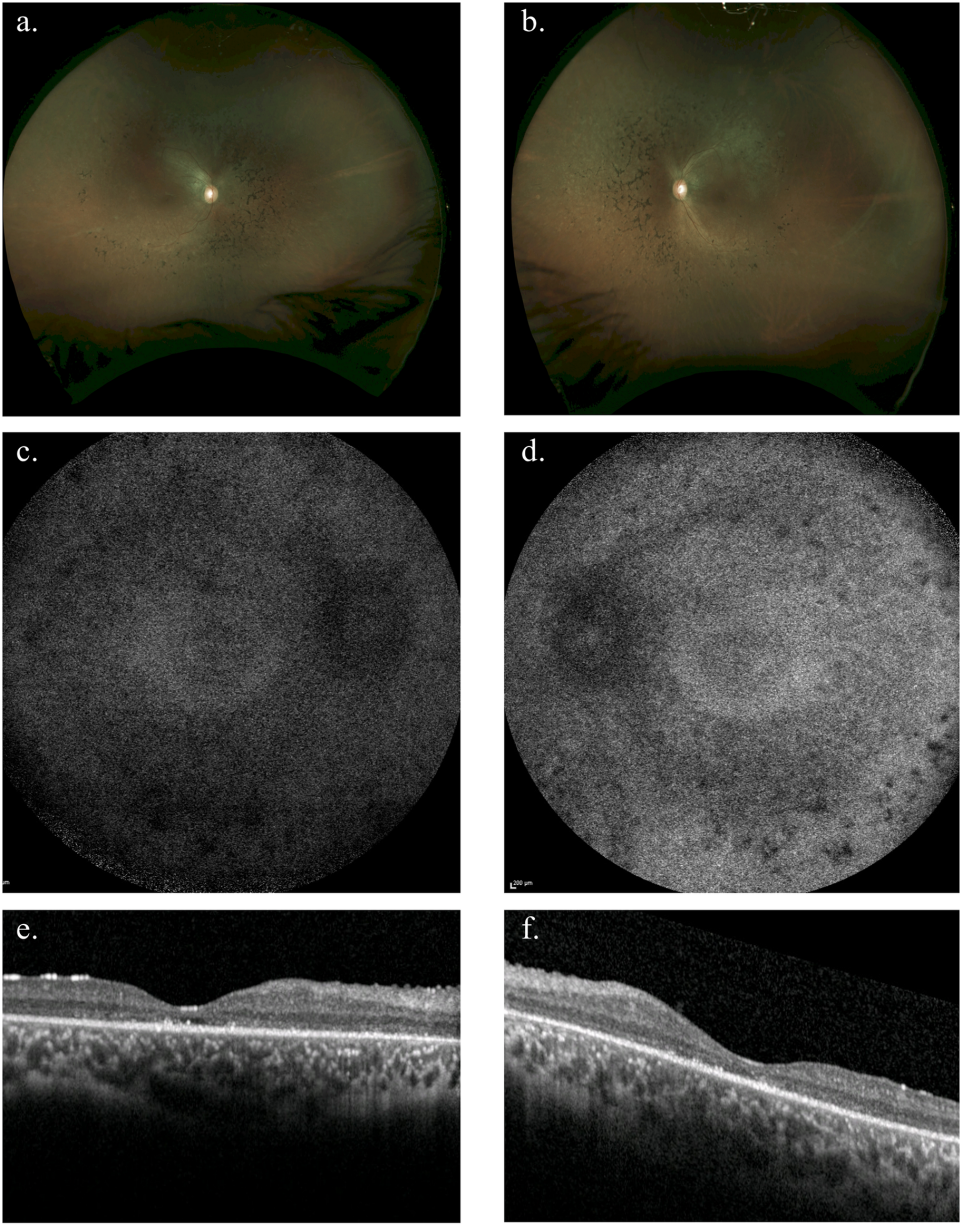
Fundus photographs and optical coherence tomography (OCT) for the affected individual in family 8. Colour fundus photographs (a and b: OD and OS respectively) revealed attenuated retinal vessels, mid-peripheral coarse pigment clumping and white dots at the level of the retinal pigment epithelium. 55 degree fundus autofluorescence imaging (c and d: OD and OS respectively) show widespread loss of autofluorescence more marked over the pigment clumps in the mid-periphery. Optical coherence tomography (OCT) (e and f: OD and OS respectively) demonstrated loss of outer nuclear and photoreceptor layers throughout the macula with occasional small foci of retained photoreceptors. This individual presented with a history of night blindness from 1–2 years of age and an intermittent divergent squint with long-standing photophobia. Presenting visual acuity was 6/9 in each eye and at last review at age 18 years had deteriorated to 6/60 each eye with severely restricted visual fields to less than 15 degrees to confrontation. There was a myopic astigmatic refractive error of right 0.25/-1.25 x 21° and left 0.25/-2.25 x 160°. Electrophysiology performed at age 7 was unrecordable.

Affected individuals demonstrated broadly consistent findings typical of arRP. These include typical retinal findings of pale optic discs, retinal vessel attenuation, and intra-retinal pigment migration on fundoscopy, typical clinical presentations for arRP with night blindness being the initial symptom in the first or second decade of life in all affected individuals. (Figs 2 & 3). ERGs when performed as adults were non-recordable (Fig 2).

The p.D25E mutation in CLCC1 was predicted to be damaging by bioinformatic analysis using Polyphen2 (HumDIV, 1, HumVar, 0.998) [9], not tolerated by SIFT (0) [10], and predicted to be deleterious by Condel (0.546) [11]. It is absent from >200 ethnically matched control chromosomes, and the dbSNP, and 1000 Genomes databases, although it is present in 13 individuals in the Genome Aggregation Database (gnomAD: 12 heterozygotes and a single homozygote in the South Asian population, allele frequency = 0.0004553 in South Asians, 0.000057 overall, (http://gnomad.broadinstitute.org/variant/1-109492985-G-T). It has not been possible to establish whether the homozygous individual had any signs or symptoms of RP.

The p.D25 residue is conserved across 22 species from human to zebrafish, with the surrounding amino acids being similarly highly conserved (Fig 1b). Possible alteration of transcription factor binding sites by the c.75C>A variant was also examined, and three potential sites, for an RXR heterodimer, a sterol regulatory element binding protein, estrogen related receptors, and GLI zinc finger family, were predicted to be disrupted while one, for SOX/SRY factors, was newly created (S3 Table). Other identified changes in the linked interval were either known SNPs or noncoding polymorphisms (S4 Table).

To identify any other putative mutations in the common 322kb critical region robustly, DNA from a single affected individual (Individual 5, Family 6) was subjected to whole genome sequencing, which failed to identify any intronic, splice site, promotor or other variants of likely functional consequence.

### Subcellular localization and molecular function studies

CLCC1 was first reported as a chloride channel localized to the endoplasmic reticulum (ER), Golgi, and nucleus [12], and contains three putative transmembrane domains (residues Val185-VLMVLLCLLCIVVLVATELWT, Leu217-LIISFLFSLGWNWMYLYKLAF, and Ile330-IPALLHLPVLIIMALAILSFC) and a signal peptide at the N-terminus (Fig 1c). To investigate possible effects of the mutation on CLCC1 function, channel activity on a microsomal membrane was measured by incorporating microsomal vesicles, prepared from wild type (WT) and mutant cells into a planar lipid bilayer with symmetrical buffers in *cis*- and *trans*- chambers, and functional channel currents were measured in microsomes (Fig 1e). The current response of the microsomes obtained from WT cells showed a large open conductance (voltage +60 mV) with rare fluctuation activity, while microsomes obtained from mutant cells presented a small current conductance with clear open/close channel activities. Higher voltage (+100mV) facilitated the gating activity in microsomes obtained from mutant cells. Current (pA) traces at differing potentials (mV) were combined to create a current-voltage plot (Fig 1f). Trendlines calculated using linear regression showed a ~three-fold decrease in the I/V conductance slope in mutant microsomes compared to WT.

While CLCC1 has been shown to function as a chloride channel [12], its role within the ER remains uncertain. To characterize this role further, interaction of CLCC1 with ER proteins was tested. Co-immunoprecipitation and western blotting showed that both WT and mutant CLCC1 interacted with Calreticulin, a major calcium binding protein in the ER lumen likely to be active in calcium storage and possibly transcriptional regulation (Fig 1d). Binding and co-localization of p.D25E mutant CLCC1 was comparable to WT. CLCC1 does not contain a KLGFFKR sequence, to which Calreticulin is known to bind, suggesting that this binding is likely distinct from Calreticulin binding to transcription factors.

**Fig 4:**
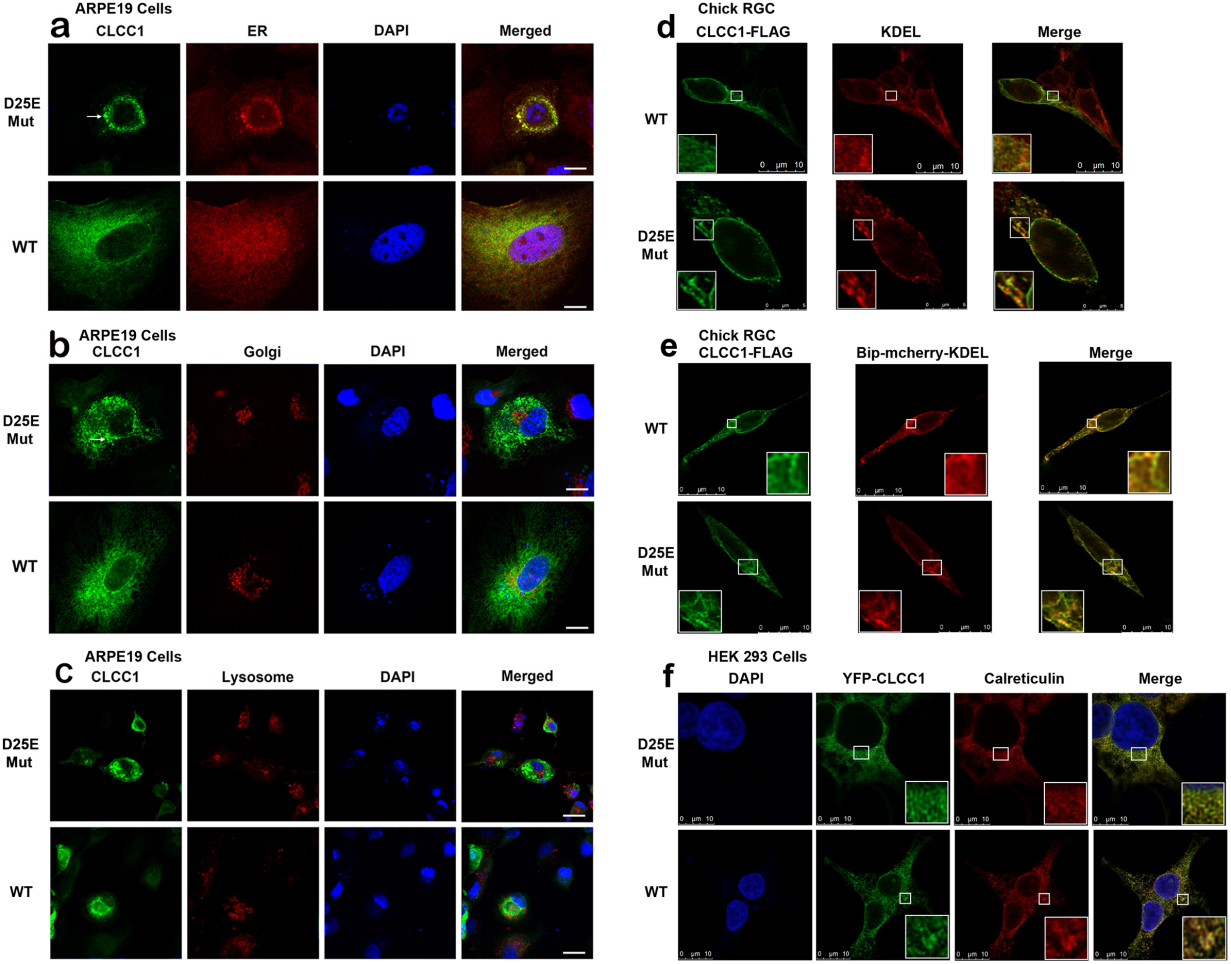
Localization of mutant and WT CLCC1 in ARPE19, Chick RGC, and HEK 293 cells by immunofluorescence. ARPE19 cells were transfected with pOTB7 expressing WT and p.D25E mutant CLCC1. (a) ER (red), (b) Golgi (red), (c) Lysosome (red), and CLCC1 (green). (d) Chick RGCs were transfected with CLCC1-FLAG (green) and KDEL (red), here CLCC1 shows extensive colocalisation with immunolabelling of the KDEL motif found in ER-associated proteins (e) Chick RGC were transfected with Bip-mCherry-KDEL (red), here the colocalization is even greater (f) HEK 293 cells were transfected with YFP-CLCC1 (green) and stained for Calreticulin (red). Cells were stained with DAPI (blue, nucleus) as well. Overlays of images from the first three columns are shown in panels labeled Merged. Scale Bar: 10 μm. CLCC1 WT and p.D25E are both shown colocalised with calreticulin. Both WT and mutant proteins localize with ER (a-f), and with neither Golgi (b) nor lysosomes (c). In the ARPE19 cells p.D25E mutant CLCC1 was concentrated in granular accumulations in the cell periphery (a-c). (d-e). The association of CLCC1 with the ER is throughout the cell, including the neurites and there is no discernible difference in the colocalisation observed with the WT or p.D25E CLCC1.

We hypothesized that the p.D25E mutation might also modify cleavage of the signal peptide, leading to accumulation of CLCC1 within the ER lumen. To compare the intracellular localization of the p.D25E mutant and WT protein, adult human retinal pigmented epithelium-19 (ARPE19) cells were transiently transfected by pOTB7 expressing WT and p.D25E mutant *CLCC1* cDNA and analyzed by immunofluorescence staining 48 hours after transfection (Fig 4). There is a dimorphic population with a subset of transfected cells expressing large amounts of WT and p.D25E mutant CLCC1 superimposed on a diffuse reticular pattern reflecting the endogenous expression of *CLCC1* as seen in the remaining untransfected ARPE19 cells (Fig 4a). From the intensity of the fluorescence, it appears that the levels of transfected CLCC1 are ~two-three times those of the endogenous molecule. Both WT and p.D25E CLCC1 proteins manifested a reticular pattern consistent with ER localization, but did not colocalize with the Golgi, lysosomes, or nucleus.

In ARPE19 cells, a proportion of p.D25E mutant protein was concentrated in granular ER accumulations and distended regions, especially the perinuclear area (Fig 4a–4c). In addition, at later time points many cells transfected with mutant CLCC1 tend to be smaller and round, although cell death was not induced (Fig 4b and 4c). These results are consistent with the p.D25E mutation leading to ER retention of increasing amounts of mutant protein over time, or perhaps simply the modified channel activity of the mutant protein. Similar distributions are seen in chick retinal ganglion cells (RGC) and HEK 293 cells (Fig 4d–4f). CLCC1 also co-localizes with calreticulin as predicted by their co-immunoprecipitation.

### *CLCC1* expression and knockdown

**Fig 5:**
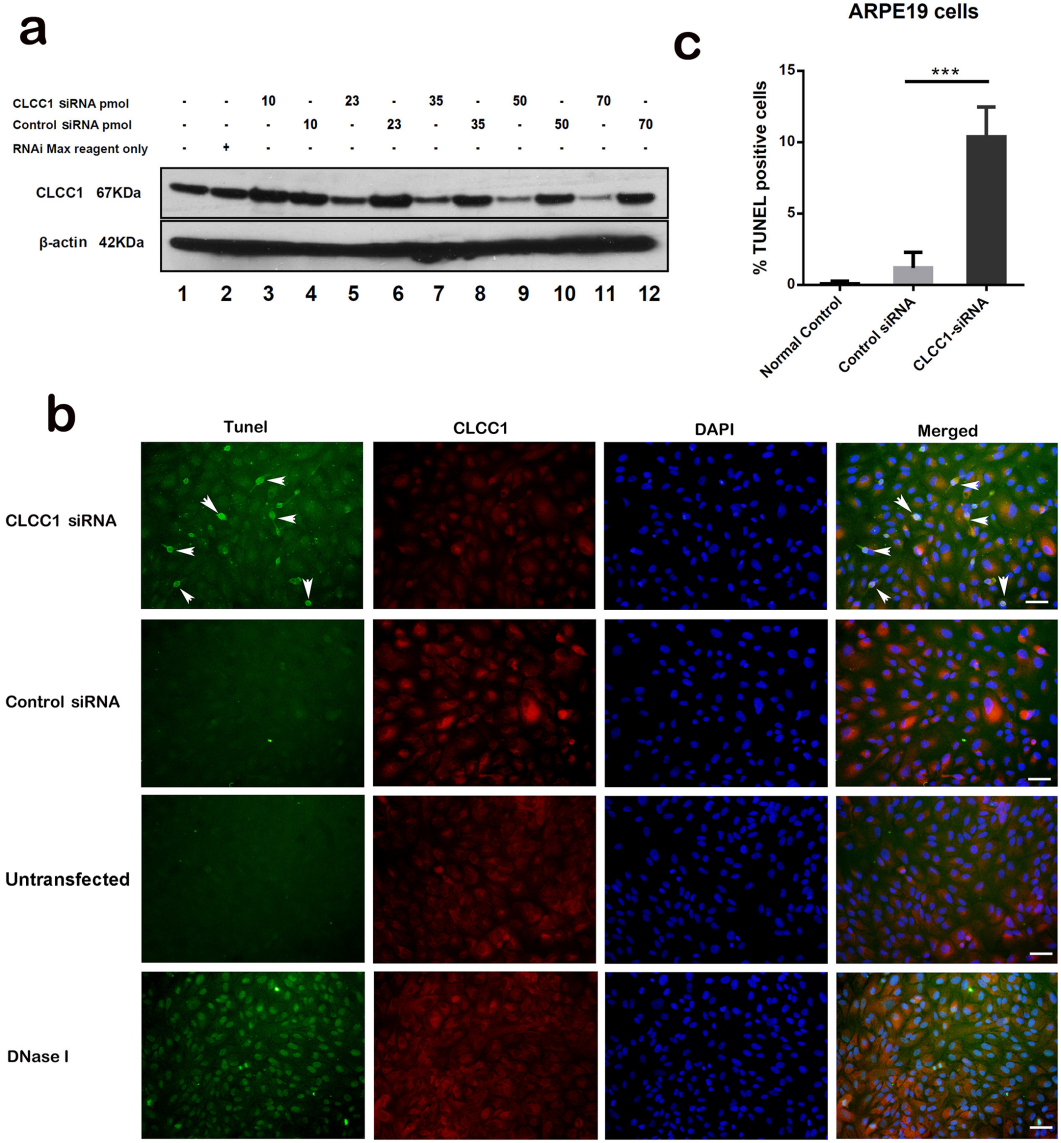
CLCC1 siRNA interference in ARPE19 cells. (a) Western Blot of SiRNA treated ARPE19 cell lysates probed with CLCC1 antibodies. Lane 1, untransfected lysate; lane 2, transfected with RNAi Max transfection reagent only; lanes 3–12, Increasing CLCC1 and control siRNA amounts from 10 pmol (lanes 3,4) to 70 pmol (lanes 11, 12). CLCC1 proteins migrate at the predicted MW of 67 kDa. The blot shows a dose dependent reduction of CLCC1 protein expression in CLCC1 but not control siRNA treated ARPE19 cells to about 20% of normal. (b) TUNEL assay after siRNA transfection. Left: TUNEL-positive apoptotic cells (green), Second: CLCC1 (red), Third: DAPI (blue, nucleus). Although there is some variation in intensity of individual cells, probably based on cell size, shape, and orientation, staining for CLCC1 is lower overall in the *CLCC1* siRNA treated cells than the control siRNA, control, or DNase 1 cells, consistent with the Western blot in Fig 5a. About 10% of *CLCC1* siRNA transfected cells were apoptotic (arrows) but there is minimal apoptosis in control siRNA or untransfected cells. DNase I treated cells were 100% TUNEL-positive. Overlays of images from the first three columns are shown in the right column labeled Merged. Scale Bar: 20 μm. **(c)** Down regulation of *CLCC1* induced apoptosis in nearly 10% of the cells (*** *P*<0.0001, *t* = 14.63) as compared to approximately 1% of cells treated with the control siRNA and less than 1% of untreated cells.

The above findings suggest two possible mechanisms for pathogenesis of the retinal degeneration. First, ER retention of CLCC1 could lead directly to an unfolded protein response (UPR) and subsequent apoptotic cell death [13]. In this regard, a role for ER stress in photoreceptor degenerations recently has been demonstrated in a number of genetic models [14–16]. Alternatively, lack of CLCC1 physiological function as a chloride transporter could be the primary contributor to the pathogenesis, perhaps by causing ER dysfunction. To differentiate these possibilities, endogenous CLCC1 protein expression in ARPE19 cells was reduced ~80% by *CLCC1* siRNA treatment (Fig 5a). TUNEL assays showed that siRNA knockdown of CLCC1, which would not be expected to cause an UPR on the basis of misfolded CLCC1, induced apoptosis in ~10% of cultured ARPE19 cells (Fig 5b and 5c). In contrast, TUNEL-positive ARPE19 cells comprised ~1% of control siRNA-treated cells and <1% of untreated cells.

**Fig 6:**
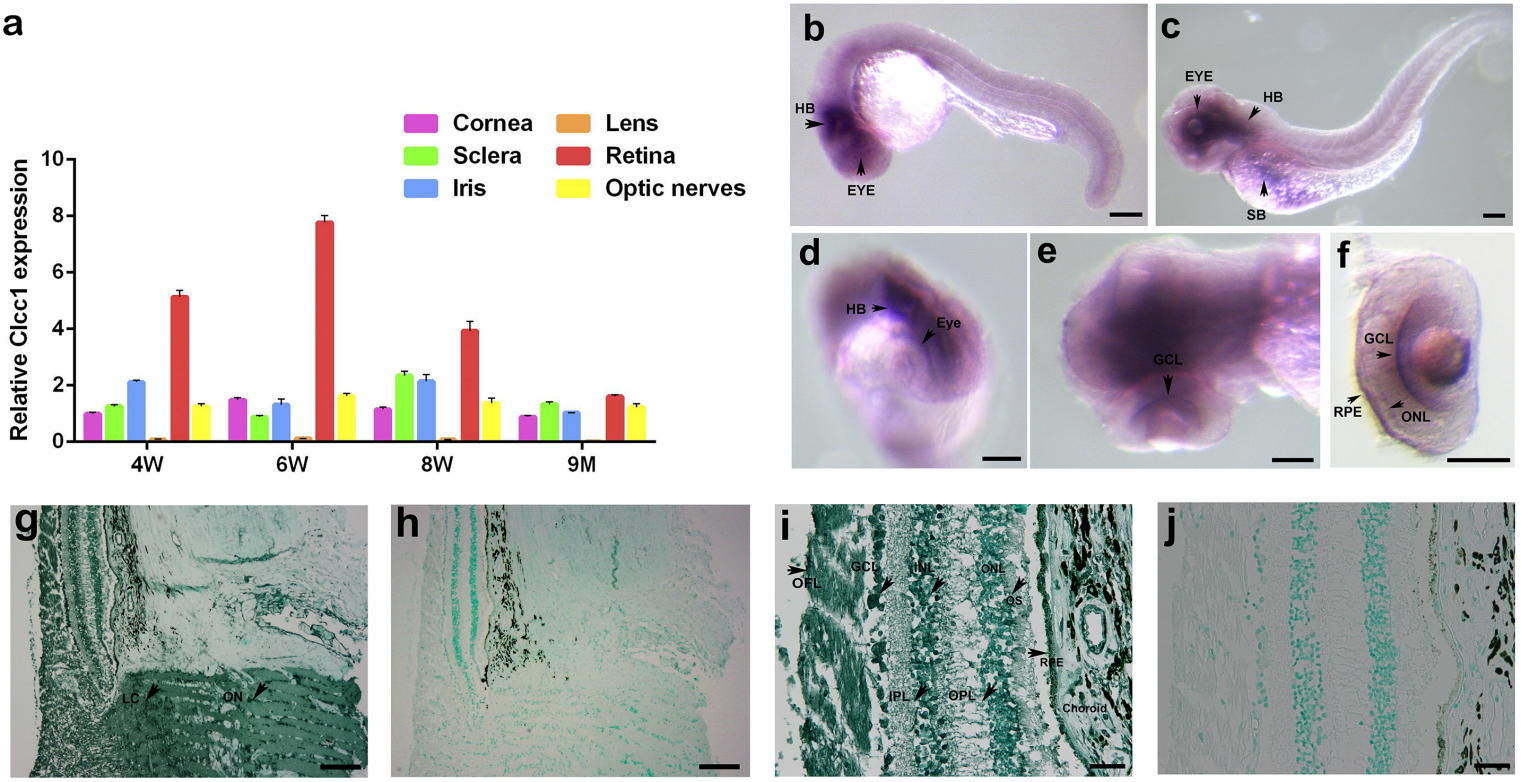
Relative expression of *Clcc1* in mouse eye tissues at various ages, distribution of *clcc1* mRNA in the zebrafish, and CLCC1 protein in the human retina. (a) Expression of *Clcc1* mRNA in the cornea, lens, iris, optic nerve, and retina by qRT-PCR at different ages. Values represent the mean (± SD) on an arbitrary scale (y axis) and were calculated from at least three independent experiments. While *Clcc1* is expressed in all tissues tested, ocular expression is greatest in the retina and least in the lens. (b-f) *In situ* hybridization of clcc1 probes in zebrafish. clcc1 Is expressed widely in zebrafish. Staining with a digoxigenin-labeled cRNA probe shows a strong signal (black arrows) in the hindbrain (HB), swim bladder (SB), and eye at 1 dpf (b, c), and in the ganglion cell layer (GCL), outer nuclear layer (ONL), and retinal pigmented epithelium (RPE) at 3 dpf (**d**, **e** and **f**); OS. Scale Bar: 100 μm. **(g–j)** IHC of formalin fixed and paraffin embedded human retinal sections demonstrated CLCC1 is expressed extensively in the retina and optical nerves. High magnification **(g,i)** shows more intense CLCC1 staining (arrow) in the lamina cribrosa (LC), optic nerve (ON), ganglion cell layer (GCL), inner nuclear layer (INL), outer nuclear layer (ONL) and retinal pigmented epithelia (RPE) in the retina (counter stain is methyl green). Scale Bar: **g, h**, 50 μm; **i, j**, 20μm.

*CLCC1* is widely expressed across the human body (NCBI: https://www.ncbi.nlm.nih.gov/gene/23155), highly expressed in the mouse retina, and moderately expressed in the iris, optic nerve, sclera, and cornea (Fig 6a). *Clcc1* expression in mouse retina increased progressively from four weeks of age, peaking at six weeks and decreasing from eight weeks to nine months, when retinal expression drops to levels similar to those found in other ophthalmic tissues. This is consistent with *Clcc1* expression being important for continuing retinal development at 1–6 weeks of age. In developing zebrafish larvae *clcc1* mRNA was diffusely expressed throughout the body. At 1 day post fertilization (dpf) expression was especially high in the hindbrain (HB), swim bladder (SB), and the eye (Fig 6b and 6c). All signals strengthened at 3 dpf (Fig 6d), at which time the ocular signal was not homogenous, being most prominent in the retina, and within the retina strongest in the ganglion cell layer (GCL), outer nuclear layer (ONL), and retinal pigmented epithelium (RPE), (Fig 6e and 6f). Immunohistochemistry (IHC) in the normal adult human eye demonstrated *CLCC1* expression extensively in the retina and optic nerve (Fig 6g–6j), indicating a physiological role of CLCC1 in retinal function. Within the retina, CLCC1 staining is more intense in the lamina cribrosa (LC), optic nerve (ON), ganglion cell layer (GCL), inner nuclear layer (INL), outer nuclear layer (ONL) and retinal pigment epithelium (RPE).

### Knockdown and knockout of Clcc1 expression in zebrafish

**Fig 7:**
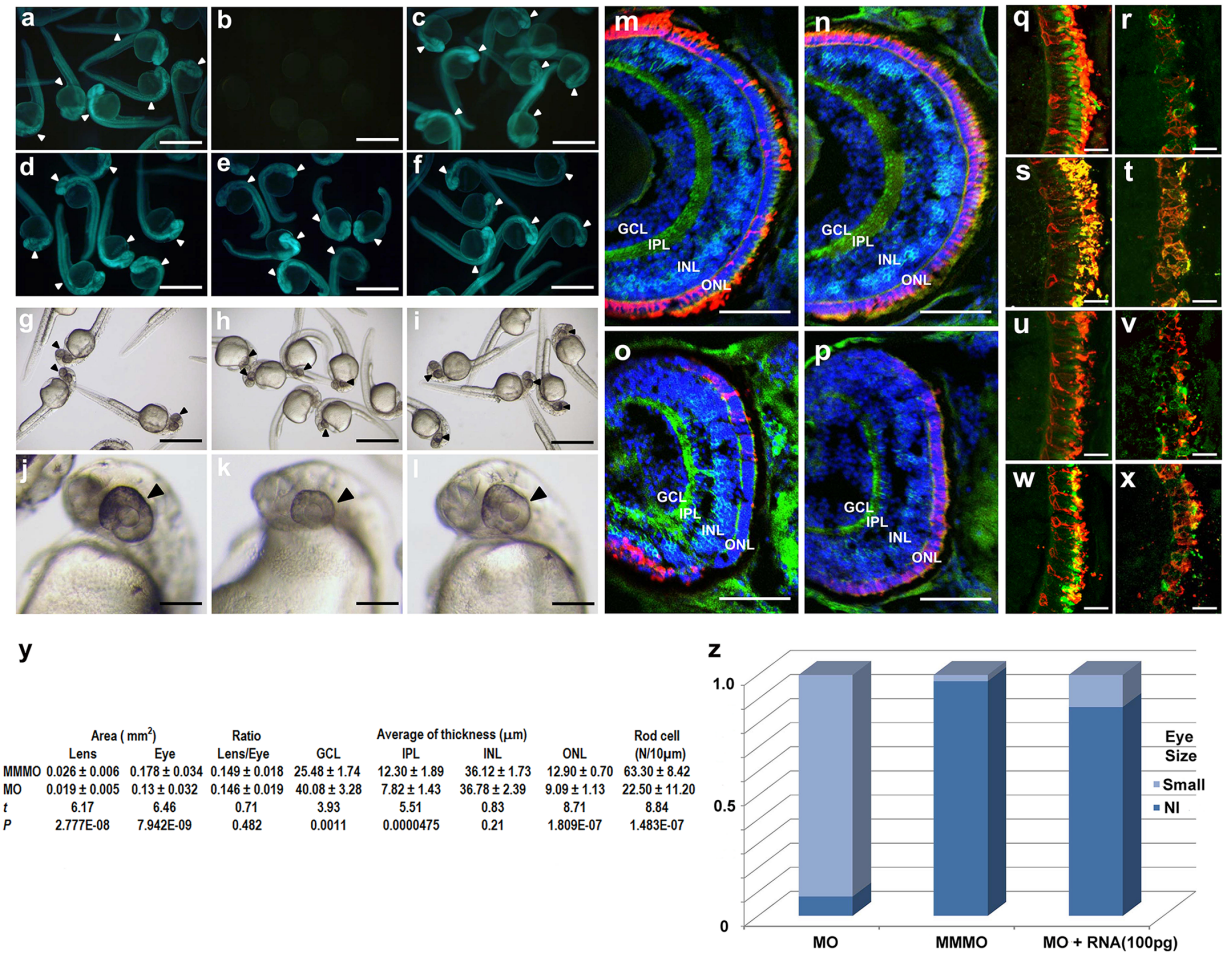
Zebrafish eye development disturbed by knockdown of *clcc1* expression. Validation: Injection of the 5’-modified EGFP (a) or the unmodified EGFP (d) gave a fluorescent signal (arrowheads). Co-injection of the *clcc1*-MO eliminated the fluorescent signal from morpholino-sensitive 5’-modified EGFP mRNA (b) but not unmodified EGFP (e). Co-injection of the MM-MO had no effect (c, f). Eye Size: Injection of the *clcc1*-MO (h, k) significantly reduced eye size (black arrows) compared to MM-MO (i, l) and buffer-injected (g, j) embryos. a-f: 24 hpf, (g-l): 36 hpf. Retinal frozen sections: from 4 dpf MM-MO- (m, n) and *clcc1*-MO-injected (o, p) embryos stained for PKCß1 (bipolar cells, green), Zpr-1 (cone receptors, red, n and p), 1D1 (rod receptors, red, m and o), and DAPI (nuclei, blue). *clcc1*-MO-injected embryos show decreased thickness of ONL and IPL layers. MM-MO-injected (q, s, u, w) and *clcc1*-MO-injected (r, t, v, x) embryos were stained with anti-blue opsin (q, r, green), anti-green opsin (s, t, green), anti-red opsin (u, v, green), or anti-UV opsin (w, x, green), and 4D2 (all, Rhodopsin, rods, red). All photoreceptors in *clcc1*-MO-injected embryos show reduced staining and damaged photoreceptor cell structure, with the greatest decreases in blue and green opsin cones. m-x: 4 dpf. Scale Bar: m-p: 50 μm, q-x: 10 μm. Comparison of eye size and retinal layers: (y). GCL = ganglion cell layer, IPL = inner plexiform layer, INL = inner nuclear layer, and ONL = outer nuclear layer. Proportions of embryos with eye size phenotype: (z) with *clcc1*-MO injection and rescue by coinjected *clcc1* WT mRNA. Lens and eye areas are given in mm2, and retinal thicknesses are given in μm. Forty *clcc1*-MO-treated embryos and 41 MM-MO-treated embryos were analyzed.

Expression of *clcc1* mRNA in the early developing zebrafish eye suggested that this might be a good model in which to explore the roles of WT and p.D25E *clcc1* in eye and retinal development. The functional role of *clcc1* in the developing zebrafish retina was evaluated using morpholino oligonucleotide (*clcc1*-MO) knockdown of *clcc1* in zebrafish embryos. Initially, specificity of the *clcc1*-MO was validated by demonstrating inhibition of translation of EGFP mRNA fused to a morpholino-sensitive sequence at its 5’-end (5’-modified EGFP) by injection of a *clcc1*-morpholino, but not a mismatch morpholino (MM-MO). In contrast, translation of unmodified EGFP mRNA was not affected by injection of either *clcc1* or MM-MO (Fig 7a–7f).

Embryos treated with the *clcc1*-MO showed normal development until 24hrs post fertilization (hpf). After 36 hpf *clcc1*-MO embryos showed reduced eye size (0.13 vs. control 0.178 mm^2^, P = 7.942x10^-9^), which was not seen in MM-MO or buffer control embryos (Fig 7g–7l and 7z). Interestingly, lens size was decreased proportionately (0.019 vs. control 0.026mm^2^, p = 2.8x10^-8^), so that the lens/eye ratio was unchanged (0.146 vs. control 0.149, p = 0.48). The inner plexiform layer (IPL), and ONL (7.82 vs. control 12.30μm 0 = 4.75 x 10^−5^ and 9.1 vs. control 12.9μm, 0 = 1.8x10^-7^) of *clcc1*-MO-treated larvae were significantly thinner in the *clcc1*-MO-treated than MM-MO-treated larvae (Fig 7m–7p and 7y). In contrast, the INL showed little change (36.78 vs. control 36.12μm, p = 0.21) and the GCL increased somewhat in apparent thickness (40 vs. control 25.48μm, p < 0.0011), The *clcc1*-MO-treated larvae showed a reduced number of rod cells (22.5 vs. control 63.3, p = 1.5x10^-7^). Those rod cells present were visible for the most part only at the retinal margin, a region of persistent neurogenesis in fish, and even at the margin, rod cells often showed abnormal morphology or were pyknotic (Fig 7m and 7o). Cone opsin staining was also somewhat decreased, and disrupted photoreceptor cell bodies were apparent (Fig 7n, 7p and 7q–7x). Specificity of the MO effect was confirmed by co-injection of *clcc1* RNA in 1-cell embryos with the *clcc1*-MOs. Rescue of the normal eye size phenotype is almost complete (87%) with co-injected WT mRNA suggesting that the observed phenotype is specifically caused by knockdown of endogenous Clcc1 protein levels (Fig 7z).

**Fig 8:**
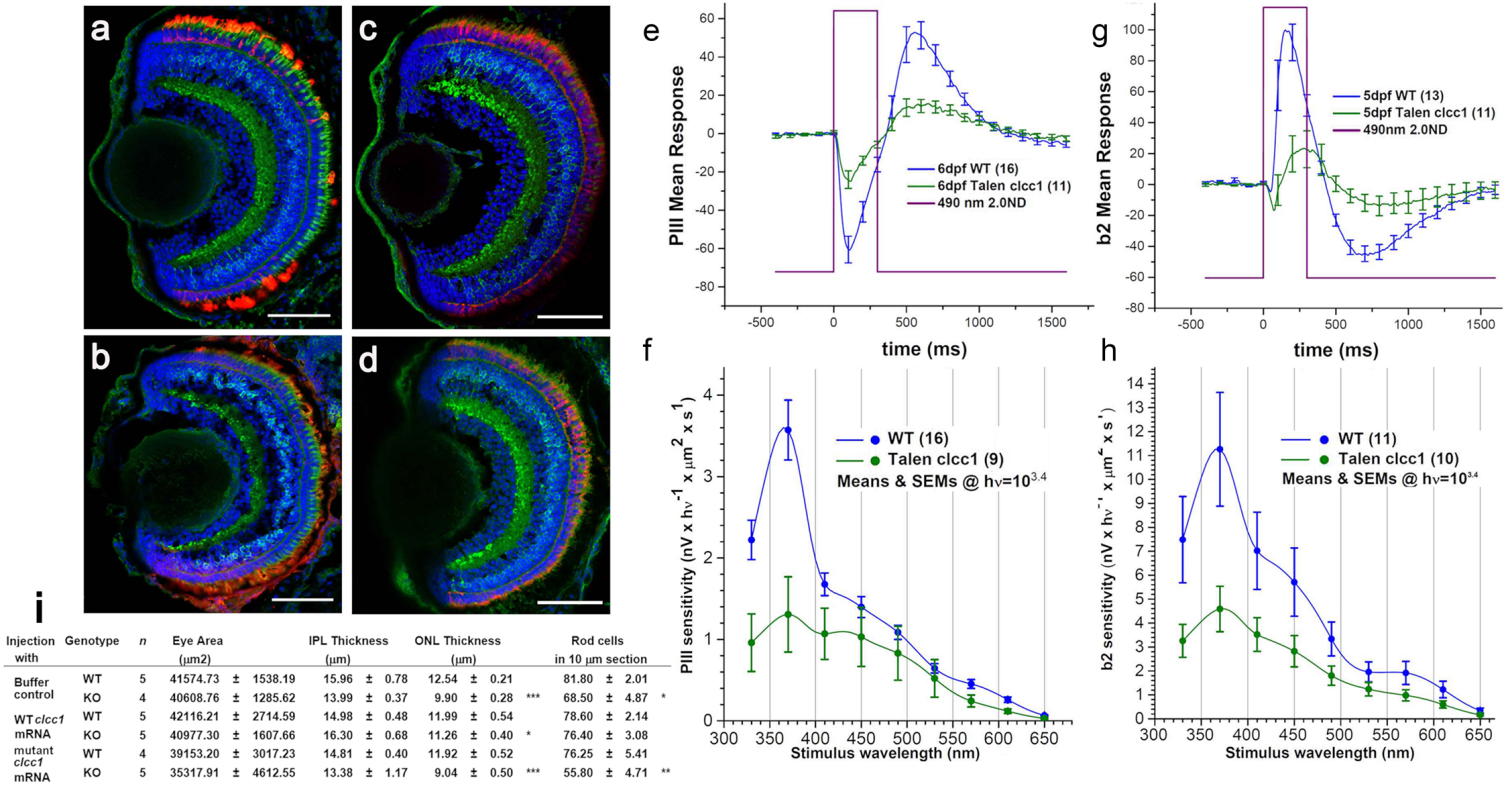
Retinal morphology and function is damaged in TALEN *clcc1*-KO zebrafish. (a-d) Merged photographs of frozen retinal sections prepared from the heads of 5 dpf larvae. Merged photos of frozen sections from KO (b, d) and WT (a, c) embryos stained for PKCß1 (bipolar cells, green), 4D2 (rod receptors, red, a and b), Zpr-1 (cone receptors, red, c and d), and DAPI (nuclei, blue). *clcc1* KO embryos show destruction of the rod photoreceptor layer (b) compared with WT (a). While somewhat better preserved than in the morpholino *clcc1* knockdown embryos, cone photoreceptors and the other retinal layers also appear decreased. (e) No background, 20mM sodium aspartate, mutant cone responses are 50% depressed relative to WT. (f) PIII 6dpf Talenclcc1 vs WT. Raw means and SEMs (all spectra). Dark adapted, no background. Cone spectral sensitivity is depressed about 60% in mutants. (g) No background, 50μM CNQX. For signals from ON bipolar cells, which are 2X more sensitive than cone signals, TALEN *clcc1* mutant responses decrease by over 50%. Stimuli are saturating at 490nm. (h) b2 5dpf Talenclcc1 vs WT. Raw means and SEMs (all spectra). Dark adapted, no background. ON bipolar cell spectral sensitivity is depressed over 50% in mutants. Sensitivity axis is in units of nV per quantum as calculated from the amplitude of responses to constant quanta stimulation across the spectrum (Eq 1). The quanta level of 2500 hν·μm^−2^·s^−1^ at the cornea is below semi-saturation for all cone types. (i) coinjection of zebrafish embryos with WT but not p.D25E mutant *clcc1* mRNA can rescue the KO phenotype. *p 0.022 vs. WT injected with buffer control, p = 0.0082 vs KO injected with p.D25E mutant *clcc1* mRNA, ** p = 0.00095 vs. WT injected with buffer control *** p > 0.00012 vs. WT injected with buffer control.

In order to confirm further the specificity of the phenotypic effects of the *clcc1* mutant in zebrafish larvae and to characterize their phenotypic effects a transcription activator–like (TAL) effector nuclease (TALENs) [17–20] was used to produce a 7 bp deletion in exon1, (c.100_106het_delAATGATG, p.N34Rfx*9) of *clcc1* at one allele (*clcc1*^+/TALEN^). The homozygous *clcc1*^-/-^ genotype mutation was lethal at around 11 dpf with no *clcc1*^-/-^ larvae detected at 15 dpf, suggesting that Clcc1 plays an essential role in vertebrate development. This finding is also consistent with the observation that the p.D25E *CLCC1* mutation inhibited channel activity only partially, allowing relatively normal development in extraocular tissues. Similar to MO knockdown fish, 5 dpf *clcc1*^-/-^ KO larvae show abnormalities in the various retinal layers including the IPL, the ONL, the rod photoreceptor layer (Fig 8a–8d), and a somewhat less severe effect on cones. When *clcc1*^-/-^ KO zebrafish embryos are injected with WT and p.D25E mutant *clcc1* mRNA, the WT but not mutant mRNA is able to reverse changes in both the ONL thickness and rod cell numbers (Fig 8i). While the ONL thickness did not return to the values in the WT injected with buffer control (11.26 ± 0.4 vs. 12.54 ± 0.21, p = 0.22), it is well above the KO value of 9.9 ± 0.28, p = 0.00819, and that of the KO injected with p.D25E mutant c*lcc1* mRNA (9.04 ± 0.50, p = 0.0082). It should be noted that MO and TALENS KO eyes are separate approaches examined on different equipment and by different investigators, so that while changes relative to controls are consistent, the absolute values for some of the measurements vary somewhat.

The TALENS induced *clcc1* mutant also allowed confirmation of the functional effects of absence of *Clcc1* expression on the mouse retina. The cone responses (PIII) for 6dpf WT and TALEN *clcc1* mutants were isolated with Na Aspartate, which blocks post-synaptic ERG signals arising from inner retinal neurons. Stimuli were saturating at 490 nm. Cone PIII ERGs showed a 50–60% depression of both cone amplitudes and sensitivity in *clcc1* KO zebrafish relative to WT fish at 6 dpf (Fig 8e and 8f). ON bipolar responses (b2) were isolated by the AMPA/KA antagonist CNQX, which blocks excitation for OFF bipolar, horizontal, amacrine, and ganglion cells (Fig 8g and 8h). ON bipolar cell spectral sensitivity was reduced by 50% in 5 dpf mutants (Fig 8g and 8h). Thus, the structural disarray and degeneration seen in *clcc1* KO fish was accompanied by correspondingly decreased function of the cone system.

### Ocular characteristics in knockout of Clcc1^+/-^ expression in mice

**Fig 9:**
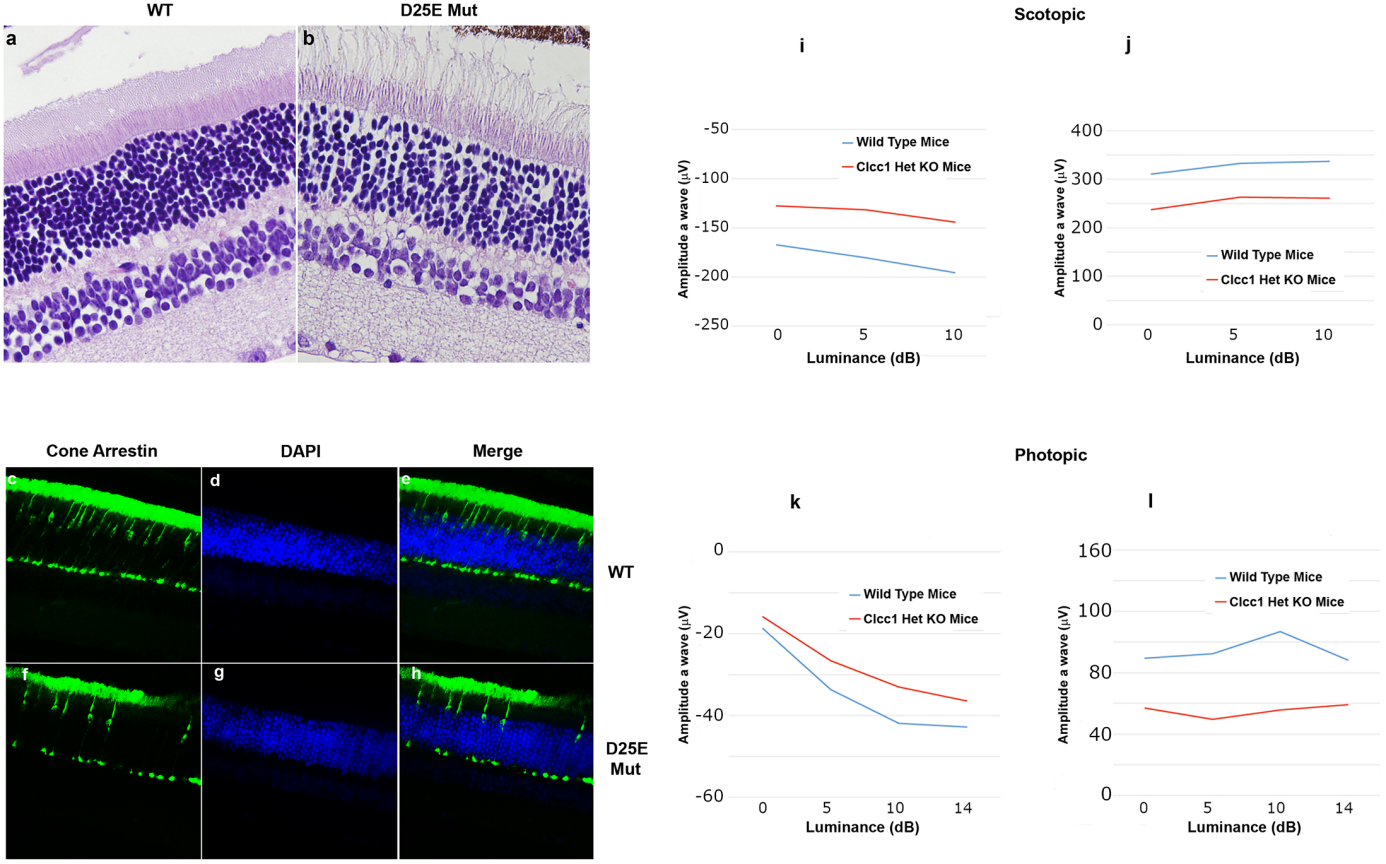
Effects of heterozygosity for *Clcc1* KO on mouse retinas. Hematoxylin & eosin staining of (a) WT and (b) *Clcc1*^*-/+*^ knockout 7-month-old mouse retinas. While the overall structure of the retina is preserved, staining revealed decreased cell density in the outer and inner nuclear layers, as well as the outer and inner plexiform layers as well as structural disarray of the photoreceptor layer in *Clcc1* KO heterozygous compared with the WT mice. (c-h) Immunostaining of cone arrestin in WT (c-e) and heterozygous *Clcc1* knockout (f-h) mice. The WT mice exhibit normal cone photoreceptors staining pattern while *Clcc1* heterozygous KO mice revealed reduced number of cone photoreceptors. (i-l) Electroretinography of WT and *Clcc1* knockout heterozygous mice show approximately 20–50% decreases in the amplitude of both the scotopic and photopic a and b wave amplitude responses in *Clcc1* KO heterozygous mice compared to WT at all levels of luminance.

To gain further insight into the effects of loss of CLCC1 function on the retina, the vision and retinal morphology of *Clcc1* KO mice were investigated. A lack of *Clcc1*^-/-^ KO mice in intercrosses of *Clcc1*^-/+^ KO mice suggested that total lack of Clcc1 activity was embryonic lethal, so the effect of heterozygosity for the *Clcc1* KO on the retina was studied (Fig 9). Based on p.D25E channel activity (Fig 1e/1f), *Clcc1*^-/+^ KO mice would be expected to display ~half the channel activity of WT, and slightly less than twice the activity of p.D25E mutant homozygotes. No abnormalities in their extraocular phenotypes have been described. Retinas of the *Clcc1*^-/+^ KO mice showed disarray and thinning of the photoreceptor layer with cell dropout in the outer and inner nuclear as well as OPL and IPL (Fig 9a and 9b), although the thickness of the retina overall was maintained. Dropout of cone cells is confirmed in Fig 9c–9h, where immunohistochemistry to cone arrestin displays ~half the cone density of WT. These morphological findings were reflected in the ERGs of the *Clcc1*^-/+^ KO mice, as the scotopic a-wave and b-wave amplitude responses are both depressed in *Clcc1*^-/+^ KO mice compared with WT mice suggesting degeneration of rods and secondary order neurons. Moreover, *Clcc1*^-/+^ KO mice exhibited a significant decreased amplitude of the photopic b-wave compared with WT mice, indicating possible dysfunction of the cone photoreceptors response and consistent with the decreased density of cones seen with immunohistochemistry (Fig 9i–9l).

## Discussion

Here we present extensive genetic, clinical and functional datasets investigating a *CLCC1* mutation as a cause of arRP. Our genetic findings of seven extended Pakistani Punjab families, as well as an eighth British-Bangladeshi family with likely similar ancestral origins, are notable and consistent with regards both to linkage, and inheritance of homozygosity of a 322 kb interval of chromosome 1p13.3 defining the critical region and providing conclusive genetic support. WES, WGS, and dideoxy sequencing of this interval identified only a single deleterious sequence alteration in the putative chloride channel CLCC1 as the likely cause of the condition. This change could theoretically affect binding of transcription factors, some of which are known to be active in the retina. However, the distance from promoter regions of *CLCC1* and nearby genes, as well as the early onset and severity of arRP in these families, would argue against effects on a single transcription factor binding site being the pathogenic mechanism rather than functional effects on the CLCC1 protein itself.

Despite the extremely low frequency of the p.D25E sequence change in the gnomAD database (http://gnomad.broadinstitute.org/about), a single homozygote is present. The gnomAD database, the successor to the ExAC database, is an aggregation of exomic and genomic DNA sequences from multiple disease specific and population projects [21]. While gnomAD have made every effort to exclude individuals with severe pediatric diseases, the database description clarifies that some may still be included, although likely at a frequency equivalent to or lower than that seen in the general population. In addition, despite symptoms and ERG signs being detectable at an early age in many [1], night blindness is present in only 61% of arRP patients by age 20 [22] and the median age of onset of diagnosis is 40y [3]. The late median age of diagnosis makes it likely that some people clinically affected remain undiagnosed within the gnomAD cohort. Evidence of this exists within other well-described arRP genes; the twice reported Thr2883LysfsTer4 frameshift *EYS* gene mutation is also present in a single homozygote in gnomAD. Thus, while it remains impossible to clarify the affectation status regarding RP of the single c.75C>A *CLCC1* homozygote present in the gnomAD database, the weight of evidence stemming from the expansive and complementary datasets presented here provide clear and strong evidence that the CLCC1 p.D25E variant is highly likely causative of this condition. Notably, there are no homozygous loss of function alleles impacting all CLCC1 isoforms present in the gnomAD database, and the low frequency of variants (all below 0.001) makes compound heterozygotes unlikely indicating that homozygous null alleles are likely lethal in humans as well as mice and zebrafish.

The important role of CLCC1 for maintaining normal retinal structure and function is illustrated by the human genetic and cell culture data, as well as the retinal morphology and ERGs in zebrafish and *Clcc1*^-/+^ KO mice. The pathogenicity of p.D25E *CLCC1* in retinal development and homeostasis is illustrated by the functional assessment of WT and p.D25E mutant CLCC1 and inability of p.D25E mutant mRNA to rescue the phenotype in *clcc1* KO zebrafish, both of which suggest that it might be a hypomorphic allele. The time course of *Clcc1* expression in mouse retina suggests a greater requirement for CLCC1 channel activity in the early postnatal period, at which time retinal cytogenesis has ended while changes in cell volume and especially the thickness of various retinal layers continue to occur, with the plexiform layers increasing and the nuclear layers decreasing in thickness and rearranging internally [23]. Our observations strongly suggest that the p.D25E *CLCC1* mutation decreases the channel activity of CLCC1 in the ER membrane, and possibly causes accumulation of mutant CLCC1 in the ER lumen, both of which would likely decrease functional CLCC1 cellular levels. The p.D25E mutation in *CLCC1* could cause disease through misfolding of the CLCC1 protein and inducing the UPR. However, in this case a single mutant allele with this gain of a deleterious effect might be expected to cause disease, resulting in an autosomal dominant inheritance pattern. In addition, apoptosis in cultured RPE cells is induced by treatment with CLCC1 siRNA, which suggests that decreased levels of functional CLCC1 alone are sufficient to cause cell death and disruption. Furthermore, decreased chloride channel function of mutant CLCC1, and failure of the mutant mRNA to rescue the KO phenotype, support lack of channel function as the underlying pathology of RP in these families. In addition, retinal morphological abnormalities and decreased photopic and scotopic ERG amplitudes in *Clcc1*^-/+^ KO mice support the critical requirement for full activity of CLCC1 for retinal function and perhaps development and structural integrity.

While *CLCC1* undergoes alternate splicing with four known isoforms, the c.75C>A (p.D25E) mutation lies in exon 2, common to all isoforms. *CLCC1* was first identified as the Mid-1-related chloride channel (*MCLC)* by homology to *Mid-1*, a stretch-activated channel in *Saccharomyces cerevisiae*, and localized to the ER, Golgi, and nucleus [12]. We confirmed localization of CLCC1 protein to the ER in ARPE19 cells, but not to the Golgi or nucleus. This may be explained by the use of a FLAG-tagged protein that might conceivably alter localization. An equally likely explanation could be that CLCC1 localization differs in various cell types, which would be consistent with nuclear localization of CLCC1 only being seen at particular stages of the differentiation of spermatogonia into spermatozoa during spermatogenesis.

Broad expression of *CLCC1* throughout the body, as well as embryonic lethality of *Clcc1*^-/-^ KO mouse and zebrafish models, begs the question of why affected individuals in these families have isolated RP without any discernable systemic signs or symptoms. While the reason for the retinal specificity of the phenotype is currently unclear there is ample precedent for isolated retinal degeneration occurring as a result of higher retinal requirements for widely expressed proteins, including components of the spliceosome (PRPF31 [24], PRPF8 [25], PRPF3 [1,26], SNRNP200 [27], PRPF6 [28], and PRPF4 [29],) and transcription factors (ZNF513) [30]. Thus, the occurrence of RP alone in these families is consistent with the hypothesis that decreased ER membrane levels of CLCC1 might be sufficient for most tissues, but not for the higher requirements of the retina, resulting in isolated arRP. In addition, high levels of *CLCC1* retinal expression and especially in the photoreceptors and RPE, are consistent with *CLCC1* mutation causing a retinal degeneration as is the finding that knocking out CLC3, another endosomal chloride channel, results in degeneration of the retina and hippocampus [31].

Recently Jia et al. identified a retrotransposon insertion in the *Clcc1* gene as a cause of autosomal recessive progressive cerebellar granule cell death and peripheral motor axon degeneration in mice [32], although they did not comment on retinal morphology or function. Patients with the p.D25E *CLCC1* alteration showed no signs of cerebellar or peripheral nerve dysfunction on examination or compatible symptoms by history, although it is possible that subtle subclinical findings were not recorded. Besides the different phenotypes, there were additional differences in the two systems. siRNA mRNA *CLCC1* knockdown in cultured retinal pigment epithelial (RPE19) cells induced apoptosis, while knockdown in embryonic kidney (HEK 293T) cells did not induce apoptosis by itself, but increased susceptibility to agents inducing ER stress. This may be consistent with greater sensitivity of retinal cells to decreases in CLCC1 function causing isolated retinal degeneration. It is interesting to note that while lack of Clcc1 is embryonic lethal in zebrafish and mice, reduced activity allows survival to adulthood in both humans and mice. However, as with the RP families, the mechanism through which the *Clcc1* mutation caused cerebellar and peripheral nerve disease in mice also appears to involve lack of *Clcc1* function causing ER stress, UPR induction, and thence apoptosis, rather than directly through the effects of unfolded *Clcc1* itself [32].

Molecular and functional characterization of CLCC1 has been minimal since its initial description, and its role in cellular metabolism, and in particular its role the retina, remains unclear. Nagasawa *et al* [12]. showed that CLCC1 is not susceptible to inhibitors of known chloride channels, suggesting that it is functionally distinct to previously described anion channel protein families, to which it bears little sequence homology. Although several proteins contain portions of the MCLC domain present in CLCC1, a Blast search of *Homo sapiens* transcripts using the *CLCC1* variant 1 mRNA displays significant similarity only to G-protein signaling modulator 2 (GPSM2), and a long intergenic non-protein coding RNA (LINC00504). However, sequence alignments between GPSM2 and CLCC1 polypeptides identify no obvious regions of homology, and a BlastP search with the CLCC1 variant 1 polypeptide identifies only CLCC1 precursors and orthologues, and two hypothetical Macaca proteins. The location of *CLCC1* alongside *GPSM2*, which codes for the putative whirlin protein complex-interacting molecule, is interesting given the role of this complex in Usher syndrome. Loss-of-function mutations in *GPSM2* have previously been associated with Chudley-McCullough Syndrome (CMCS [MIM 604213]), a rare autosomal recessive disorder characterized by sensorineural deafness, agenesis of the corpus callosum, frontal polymicrogyria, interhemispheric cyst, and ventricular enlargement. Importantly none of the non-ocular features characteristic of CMCS are present in the affected individuals presented here. Consistent with this, genome sequencing of the c.75C>A *CLCC1* haplotype identified no additional intronic, promotor of other sequence alterations in the vicinity of *GPSM2* (or in other genes in the 322kb region) that might influence functionality. While these findings do not support involvement of GPSM2 in the condition reported here, it remains unclear whether there may be any functional relationship between the two molecules that may be impacted by the p.D25E CLCC1 variant.

In conclusion, we have identified a c.75C>A, (p.D25E), mutation in the presumptive intracellular chloride channel CLCC1 as the cause of arRP in eight Pakistani families. The p.D25E mutated CLCC1 is localized to the ER in cultured cells, and siRNA inhibition of *CLCC1* expression in ARPE19 cells causes apoptosis. Knockdown of CLCC1 protein levels in zebrafish embryos with MO inhibition of translation as well as TALEN knockout of the *clcc1* gene both result in defective retinal development and photoreceptor loss. The p.D25E mutation decreases chloride channel activity, and the *clcc1* KO zebrafish phenotype is rescuable by WT but not by p.D25E mutant mRNA. Elucidating the pathophysiology of CLCC1 in arRP promises to provide insight into its biological role in retinal development and homeostasis, as well as the mechanisms underlying retinal degeneration. These findings also expand the spectrum of genetic causes of arRP, and have the potential to improve our molecular diagnostic and perhaps eventually therapeutic abilities for this form of arRP. In addition, since *CLCC1* lies in close proximity to the *ABCA4* gene, mutations in this gene may account for RP in some families in which the disease has been mapped to the region, but in which mutations in *ABCA4* have not been identified.

## Materials and methods

### Ethics statement

Human studies detailed in this manuscript were reviewed and approved by the Combined Neuroscience IRB (Protocol# 16-EI-0104), the National Eye Institute (Protocol #NEI-659), the IRB of The Institute of Biomedical and Genetic Engineering and National Centre of Excellence in Molecular Biology (Pakistan; Protocol #IRB-V1/18), and animal studies were approved by the NEI/NIH ACUC (Protocol# NEI-659) and the Johns Hopkins Animal Care and Use Committee (ACUC; Baltimore, MD, USA).

### Family and clinical data

Alternative nomenclature of families is provided at first reference for ease of cross reference with previous work by J. Fielding Hejtmancik and colleagues. Informed consent was obtained from all participating individuals, conforming to the tenets of the Declaration of Helsink, with approval by the CNS IRB of the National Institutes of Health, Bethesda, MD, and the IRBs of the National Centre of Excellence in Molecular Biology as well as the Institute of Biomedical and Genetic Engineering, Lahore, Pakistan. The diagnosis of RP was based on symptomatic night blindness beginning in early childhood, progressive loss of peripheral vision, attenuation of retinal vessels, pigment disturbance on fundus examination, and decreasing visual acuity with age. Electroretinogram (ERG) responses were recorded consistent with ISCEV standards [33] in selected cases, using commercial ERG equipment (LKC, Gaithersberg, MD, USA). Under scotopic (dark-adapted) conditions, the single bright-flash stimulus elicits a response dominated by rod activity but that also contains a cone component. The photopic (light-adapted) 30 Hz flicker stimulus elicits activity exclusively from the cone system.

### Fine mapping, linkage analysis and haplotyping

Markers were selected for fine mapping from the Marshfield database, as were the marker order and distances between markers. Two-point linkage analyses were performed using the FASTLINK version of MLINK and ILINK from the LINKAGE program package [34, 35]. RP was analyzed initially as a fully penetrant autosomal recessive trait with a disease allele frequency of 0.00001 and equal marker allele frequencies. For fine mapping allele frequencies were estimated from genotypes of 100 unrelated and unaffected individuals from the Punjab province of Pakistan. Haplotypes were constructed by observation. SNP genotyping was performed using Illumina Human CytoSNP-12v2.1 330K arrays.

### Next generation sequencing and Sanger sequencing

Next-generation whole exome sequencing was performed at the Cincinnati Children’s Hospital and Medical Center (CCHMC) core facility (Individual 12, Family 2), the University of Exeter Medical School (Individual 5, Family 6), UCL Institute of Ophthalmology (Family 8, using a previously described method) [36] and the Baylor College of Medicine Human Genome Sequencing Center (BCM-HGSC; Individual 8, Family 7 using a previously described method) [37]. Genomic DNA was captured with the Agilent SureSelect Human All Exon kit, or VCRome 2.1 design (52 Mb, NimbleGen, Cat. No. 06266380001; BCM-HGSC), according to the manufacturer’s protocol (Agilent, Santa Clara, CA). Subsequent to capture and enrichment, the paired-end library was sequenced on an Illumina HiSeq2000 Genome Analyzer (Illumina, San Diego, CA). Multiplexing of three samples per lane on a HiSeq 2000 generated an average 80 x coverage for each exome. The raw data were aligned (ELAND) and mapped (Seqmate) to the UCSC Hg19 reference; variants were called using ATLAS and annotated with the in-house-developed ‘‘Cassandra” annotation pipeline (BCM-HGSC). SNP and INDEL (insertion/deletion) calls for each sample were made according to the GATK recommendations. To confirm next generation sequencing findings, exons including over 100 bp of flanking sequence of genes in the linked region were screened by Sanger sequencing using an ABI PRISM 3130 automated DNA analysis system (Applied Biosystems) and analyzed with Seqman software (Lasergene).

Whole genome sequencing (WGS) was performed by BGI Tech Solutions (Hong Kong) on the BGISEQ-500 sequencing system.

### Preparation of microsomes

ARPE19 cells, homogenized in lysis buffer (20 mM HEPES, 10 mM KCl, 1 mM MgAc_2_, pH 7.4), were lysed by passaging through a 25 G needle with 10 passes on ice (1 pass means up and down). After mixing the lysate with an equal voluminal of 20% sucrose, the lysate was centrifuged at 7000 g/min for 10 min. The supernatant was taken and centrifuged at 16000 g/min for 90 min. Then the precipitate was resuspended with 50 mM HEPES, 150 mM KAc, 10 mM MgAc_2_, 250 mM sucrose, pH 7.3 to obtain microsomes suspension.

### Single-channel recording

Single-channel recordings in planar lipid bilayer were performed using Ionovation Compact (Osnabrück, Germany). The same buffer (5 mM Tris, 5 mM MOPS, 150 mM KCl, pH 7.0) was present in both *cis*- and *trans*-chambers, which were separated by a 25 μm thick TEFLON film with an aperture of 50–100 μm diameter. Voltage was applied across the bilayer using Ag/AgCl electrodes immersed in each chamber. The artificial membrane was formed by painting the solution of POPC/POPG (3:1) (Avanti Polar Lipids) in n-decane on the aperture, and formation was monitored by capacitance measurements. The microsomes suspension was added to the *cis*- chamber next to the bilayer. Currents were measured with a 2 kHz low-pass filter at 10 kHz sampling rate using an EPC-10 amplifier (HEKA Elektronik). Data were analyzed using Clampfit software (Axon Instruments).

### Cloning

Bip-mCherry-KDEL is based on Zurek et al., 2010 and subcloned in pcLink. Forward: 5’-AATAAGATCCATGAAGTCCTCCCTGGTGGCCGCGATGCTGCTGCTGCTCAGCGCGGCGCGGGCCGTGAGCAAGGGCGAGGAGGATAAC-3’. Reverse 5’-ATATCTGTCATAGCTCGTCTTTCTTGTACAGCTCGTCCATGCC-3’. Human *CLCC1* cDNA was cloned in pcLink. Forward: 5’-TGAAGCTGGAAAGCTTGGAC-3’. Reverse: 5’- ATTAGTCGACCTAGCCACAGGGGCTGCTGACCGG -3’.

### Chick RGC isolation

Fertilized hens’ eggs (*Gallus gallus*) were obtained from Henry Stewart & Co. Ltd., Lincs. and incubated in a humidified, forced-draft incubator at 38 °C. At E7 embryos were removed from the egg, decapitated and the retinal ganglion cell layer was collected. Cells were counted and nucleofected with the 4D-Nucleofector system (Lonza) in P3 buffer (Lonza) with 0.4ug of DNA, following the manufacture instructions. Cells were seeded on PDL/Laminin coated coverslips and cultivated in Neurobasal medium Phenol-red free, Glutamine free (Gibco), supplemented with 180 μM Hepes, 0.5mM L-Glutamine, 10U/mL penicillin/streptomycin, 2% B27 supplement. After 24h cells were fixed with a mixture of 4% Paraformaldehyde and 0.2% Glutaraldehyde for 15 min at 37°C.

### HEK 293 transfection and staining

HEK 293 cells were cultivated in DMEM supplemented with 10U/mL penicillin/streptomycin and 10% fetal bovine serum, and kept in the incubator at 37C with 5% CO_2_. Cells were transfected with plasmids using Lipofectamine LTX reagent (Invitrogen), following the manufacture instructions, and fixed in 4% PFA for 15min at RT after 24h. Coverslips were incubated with lysine buffer (PBS containing 5% horse serum; 5% goat serum; 50mM L-lysine; 0.2% Triton X-100), stained with Calreticulin 1:100 (PA3-900 Thermo Fisher) and Alexa-568 anti-rabbit. Coverslips were mounted using FluorSave (Calbichem) and analyzed at the Leica SP6 confocal microscope, supported by the Huygens deconvolution system (SVI).

### Co-immunoprecipitation and Western blot experiments

ARPE19 Cells were lysate in 20mM Tris-HCL, 150mM KCL, 0.1% Triton, 1x Protease and phosphatase inhibitors (Sigma-Aldrich) and 20mM PMSF. The Co-immunoprecipitation was performed using Dynabeads Protein G (Life Technologies) with the following protocol adjustments: beads were incubated for 1h with CLCC1 (HPA013210; Atlas Antibodies) antibody at RT, and incubated 2h at 4°C with the total protein lysate. Washing buffer contains: 1M Tris pH8.0, 1M KCl, 10% Triton X-100. Proteins were eluted in 4x NuPage Sample Buffer (Invitrogen), 10% β-mercaptoethanol. Western Blot was performed using 10% Acrylamide/Bis acrylamide gel and SDS-PAGE buffer. Proteins were immobilized on the PVDF membrane (Millipore). Antibodies (CLCC1 1:1000, Calreticulin 1:1000, GAPDH (MA5-15738, Thermo Fisher) 1:1000) were dissolved in TBST and 5% semi-skimmed milk. Membrane was stained with HRP-conjugated secondary antibody. The revelation was performed using C-Digit Li-Cor system (Li-Cor Bioscience).

### Immunohistochemistry (IHC)

Human specimens (eyes and optic nerves) were fixed in formalin and embedded in paraffin. The specimens were then sectioned through the lesions and subjected to avidin-biotin-complex immunohistochemistry as described previously [38]. Primary antibodies were against CLCC1 (Novus Biologicals, NBP1-82793), the secondary antibody was biotin-conjugated anti-rabbit IgG, and counterstaining was applied with methyl green.

### Cloning of human *CLCC1* and site directed mutagenesis

Human *CLCC1*-cDNA was cloned into pOTB7 vector (Thermo, MHS4771-99610792). PCR primers CLCC1-MutF: ATGACTGGATTGAACCCACAGACATGC, CLCC1-MutR: GCATGTCTGTGGGTTCAATCCAGTCAT were used to introduce a c.75C>A mutation into the human *CLCC1* gene. The *Dpn1* digested amplicon was transformed into chemically competent cells (Invitrogen). The mutation was validated by dideoxy DNA sequencing.

### Analysis of WT and mutant *CLCC1* expressed in ARPE19 cells

ARPE19 cells were transfected with WT and mutant *CLCC1* constructs using PolyJet (SignaGen, Rockville, MD). The cells were harvested after 48 hrs. After transfection and Western blot analysis was performed with 30 μg of reduced proteins separated on 4–12% Bis-Tris precast polyacrylamide gel (Bio-Rad, Hercules, CA). CLCC1 antibody (Novus Biologicals, Littleton, CO, NBP1-82793, Rabbit, 1:100) was used for the Western blots.

### Immunofluorescence analysis of transfected ARPE19 cells by confocal microscopy

ARPE19 cells were transfected with expression constructs using PolyJet (SignaGen, Rockville, MD). Paraformaldehyde fixed cells were incubated with primary CLCC1 antibody (rabbit, 1:200, Novus Biologicals, Littleton, CO, NBP1-82793), ER (mouse, 1:300, BD Biosciences, San Jose, CA, Anti-PDI), Golgi (mouse 1:300, BD Biosciences, San Jose, CA, anti-GM130) antibodies and Lysosome (Invitrogen, Grand Island, NY, LysoTracker Red DND-99, red) followed by washing with 1% BSA in PBS and incubation with the secondary antibody, anti-rabbit Alexa 594 and anti-mouse Alexa 488. Subsequently, washed cells were mounted with Vectashield anti-fade mounting medium containing DAPI. Immunofluorescence was visualized with a Zeiss LSM 700 laser scanning confocal microscope.

### RNA interference (RNAi) and TUNEL assay

ARPE19 cells plated at 60% confluency in 6-well plates were transfected using Lipofectamine RNAiMax reagent (Invitrogen, Grand Island, NY) according to the vendor’s instructions with 10, 23, 35, 50 and 70 pmol of CLCC1 siRNA (4390843). The cells were harvested for Western blot analysis 48 hrs. after transfection unless otherwise noted. The TUNEL assay was performed using Click-iT TUNEL Alexa Fluor Imaging Kit (Invitrogen, Grand Island, NY) in accordance with the manufacturer’s protocol. In brief, cells were fixed with freshly prepared 4% paraformaldehyde in PBS at room temperature for 20 min and permeabilized with Triton X-100 (0.25% in PBS) for another 20 min. The cells were then washed twice and incubated with 50 μl of terminal deoxynucleotidyl transferase reaction buffer (Component A) for 10 min at room temperature. The buffer was removed and the TUNEL reaction mixture containing terminal deoxynucleotidyl transferase was added, followed by incubation of the cultures in a humidified chamber at 37 °C for 60 min. After treatment, cells were washed three times with 1% BSA in PBS for 5 min each, and incubated with antibody to CLCC1 (Novus Biologicals, Littleton, CO, NBP1-82793) for 1hr followed by three washes with 1% BSA in PBS for 15 min each. Cells were then incubated with anti-rabbit Alexa 594 antibody and 50 μl of Click-iT reaction mixture (containing Alexa 488 azide) for 1 hr. The cells were again washed with 1% BSA in PBS and the cell nuclei were counterstained with DAPI for 15 min at room temperature. The coverslips were washed twice with PBS before mounting onto a slide with Vectashield mounting medium (Vector Laboratories, Burlingame, CA). Negative and positive controls were included in each experiment. For the negative control, cells were processed using a reaction mixture that did not contain terminal deoxynucleotidyl transferase. For the positive control, cells were incubated with DNase I (3 units/ml) for 30 min to induce DNA strand breaks. Labeled nuclei were detected by fluorescence microscopy on an OLYMPUS BX41 microscope. TUNEL-positive cells were counted in 21 different, random fields for each well.

### Quantitative RT-PCR of *Clcc1* in different mouse eye tissues

Total RNA was isolated from tissues or cells using Trizol reagent (Invitrogen). After Turbo DNA-free DNase treatment (Ambion, Grand Island, NY), reverse transcription was carried out with SuperScript III First-Strand Synthesis system (Invitrogen, Grand Island, NY) using 1 μg of RNA. Quantitative real-time RT-PCR was performed on a ViiA^™^ 7 Real-Time PCR System (Applied Biosystems Inc.) The PCR was performed as follows: 2 min 30 sec at 95°C for enzyme activation followed by 40 cycles of 15 s at 95°C, and 1 min at 60°C. Melting curve analysis was performed to confirm the real-time PCR products. All quantifications were normalized to 18S rRNA levels. Primer sequences used are: *Clcc1*-F:ATGGCCTTATTCAGGATGC, *Clcc1*-R:TCTCATCAGGGAGTCCAAGC.

### *In situ* hybridization analysis

The 5′-probe specific for the *clcc1* cDNA clone was amplified by PCR and cloned into the pcDNA3 vector (Invitrogen). The following primers were used to prepare *clcc1* probes (the size of the amplicon is 1192 bp): *clcc1*- ISH-PrimerF, aggtgaagctttccaagcag, *clcc1*- ISH-PrimerR, gaagatggtggctcactgg. The digoxigenin-labeled cRNA probe was synthesized by in vitro transcription of the corresponding linearized plasmids using T7 polymerase and DIG-RNA-labeling mixture (Roche). Whole mount *in situ* hybridization was performed as described previously [39].

### Fish breeding and maintenance

Zebrafish were maintained at the NIAAA animal facility, following the LMP-FO-21 protocol approved by the NIAAA.

### Morpholino injections

Translation-blocking Morpholino (MO) antisense oligonucleotides (Gene Tools) were used to knockdown *clcc1* expression in zebrafish. The MOs (1 ng) dissolved in injection buffer (0.025% phenol red, 120 mM KCl, and 20 mM HEPES, pH 7.4) were injected into egg yolks of 1–4 cell stage embryos. Clcc1-sensitive EGFP was constructed by adding the sequence, 5’- GATT (ATG)AAGCTGTCAAGCTCTTCAGTGAGCAAGGGCGAGGAGCT -3’ to the 5’-end of EGFP cDNA and cloning into the pCS2+ vector as was unmodified EGFP cDNA. RNA was synthesized using a mMESSAGE mMACHINE kit (Ambion, Life Technologies, Grand Island) and injected to one cell stage eggs before the MO injection. Note that all embryos contain faint yellowish autofluorescence in the egg yolk not derived from EGFP. The c.75C>A mutant *clcc1* mRNA was synthesized and cloned into the expression vector before being used to rescue.

### Frozen sections and immunofluorescence

Fixed embryos were equilibrated in 20% sucrose and embedded in OCT (Electron Microscopy Sciences). Frozen sections (10 μm) were produced using a cryostat (CM3050, Leica Microsystems). Sections were blocked with 5% normal goat serum and incubated with anti-PKCß1 (1:300, Santa Cruz), Zpr-1 (1:200, Zebrafish International Resource Center), 1D1(1:50), 1D4(1:1000),4D2 (1:400, Abcam) and rabbit anti opsin(anti-blue 1:250; anti-green, 1:500; anti-red, 1:500; anti-UV, 1:1,000) [30]. Slides were washed in PBS and incubated with Alexa488- or Alexa594-conjugated goat anti-mouse IgG (1:400, Invitrogen) and with DAPI for nuclear staining. Images were collected using a Zeiss LSM 700 confocal microscope. The thickness of each retinal layer and the number of cells were measured using ImageJ software. The numbers of embryos examined were *clcc1*-MO-treated (n = 40), and MM-MO-treated (n = 41) for retinal layer thicknesses (given in μm), and for rod cell numbers: *clcc1*-MO-treated (n = 8) and MM-MO-treated (n = 10) larvae. The unpaired Student’s t test statistic *t* was used for comparisons, and p < 0.05 was considered statistically significant.

### clcc1-TALEN fish generation

The synthetic TAL CLCC1_F and TAL CLCC1_R were assembled from synthetic PCR products. The fragment was cloned into TALtrunc_FokI Vector using 100% sequence verified subfragments. The resulting plasmid DNA was purified from transformed bacteria and quantified by UV spectroscopy. The TAL CLCC1_F TAL binding sequence was TGCAAAAATCCGCATTGAC corresponding to RVD (T)-NN-HD-NI-NI-NI-NI-NI-NG-HD-HD-NN-HD-NI-NG-NG-NN-NI-HD. The TAL CLCC1_R TAL binding sequence was TCATATGGATCAATCCAAG corresponding to RVD (T)-HD-NI-NG-NI-NG-NN-NN-NI-NG-HD-NI-NI-NG-HD-HD-NI-NI-NN, where '(T)' indicates that the first binding repeat is provided by the vector. Fragments encoding TAL effector repeat arrays were cloned into pEXP2-DEST expression vectors. All TALEN expression vectors were verified by Seqman software (Lasergene).

TAL CLCC1 RNAs were synthesized using the mMESSAGE mMACHINE T7 Ultra kit (Ambion). Briefly, TALEN DNA was linearized with PmeI (NEB) and purified. RNAs encoding each TALEN arm were combined and resuspended in nuclease free water at a concentration of 50 ng/ul. A mixture of 50 pg of each TALEN RNA was injected into the single cell zebrafish embryo. Fish genomic DNA was extracted from 3 days post fertilization (dpf) larva or adult fish tail fin by ZyGEM Tissue kits and PCR was performed using primers that span the target site of interest. Genomic PCR products were sequenced and the alignments were made to determine the indels at each allele using Seqman software (Lasergene). For rescue experiments the wt and c.75C>A mutant *CLCC1* and EGFP cDNAs were synthesized and cloned into the pCS2+ vector as was unmodified EGFP cDNA. RNA was synthesized using a mMESSAGE mMACHINE kit (Ambion, Life Technologies, Grand Island) and injected to one cell stage eggs before the MO injection.

### Zebrafish photopic electroretinogram (ERG)

Zebrafish electroretinograms (ERG) are the sum of light evoked extracellular field potentials generated by the aggregate of retinal cells, summing the extracellular limbs of current loops set in motion by light stimulation of all retinal cells. These produce net, light-evoked voltage changes at the retinal surface [40] culminating in a series of named ERG peaks and troughs. ERG responses were recorded *in-vitro* from perfused larval eyes using modified patch electrodes (3 μm tip) inserted through the cornea into the ocular vitreous. The spectral—sensitivity and intensity—response functions of zebrafish cones were isolated by blocking photoreceptor synapses using 20 mM L-aspartic acid (Sigma-Aldrich), which eliminates signals from inner retinal neurons [41 42]. To isolate the b2 response of ON-bipolar cells post-synaptic to zebrafish cones [43], the MEM perfusate contained 50μm CNQX (Tocris), which blocks cone AMPA/kainate synapses onto hyperpolarizing OFF-bipolar cells. MEM was equilibrated with 95%O_2_, 5%CO. Spectral characteristics of the mean PIII or b2 waveforms were averaged (n = 4) at seven stimulus intensities at each of nine wavelengths. PIII or b2 amplitudes were modeled as a summation of 4 spectrally weighted Hill functions, one each for UV (362nm), blue (415nm), green (480nm) and red (570nm) cone signals, present in varying amounts within ERG subcomponents [43]. Responses were evoked on an infrared background used for visualization, which did not alter retinal activity.

$$V=\frac{VrI}{I+\frac{Kr\text{570}}{Ar(\lambda )}}+\frac{VgI}{I+\frac{Kg\text{480}}{Ag(\lambda )}}+\frac{VbI}{I+\frac{Kb\text{415}}{Ab(\lambda )}}+\frac{VuI}{I+\frac{Ku\text{362}}{Au(\lambda )}}$$ (1)

Above, ***V*** is PIII or b2 amplitude, ***I*** is stimulus irradiance, and ***λ*** is stimulus wavelength. **V**_r_, **V**_g_, **V**_b_, and **V**_u_ are amplitude maxima for extracted cone components. **k**’s are individual cone half saturation irradiances. Fixed quantal—irradiance amplitude spectra, at 2500 hν·μm^−2^·s^−1^ incident on the cornea, are generated from fits to Eq 1. Mean spectra with SEM’s are plotted after recordings from multiple eyes.

### *Clcc1* knockout (KO) mice

*Clcc1* heterozygous KO mice with exon 7 replaced by a lacZ reporter allele [44] were purchased from Jackson Laboratories (Stock number: 027239, B6N(Cg)-*Clcc1*^*tm1b(KOMP)Mbp*^/2J) and mating of heterozygous KO mice was set to propagate the line as homozygous KO mice were embryonic lethal.

### Mouse electroretinography

Electroretinography (ERG) responses were recorded for seven-month-old *Clcc1* heterozygous KO and control mice (age- and sex- matched) using a BigShot Ganzfeld and UTAS system (LKC Technologies, USA). The use of animals in this study was approved by Johns Hopkins Animal Care and Use Committee (ACUC; Baltimore, MD, USA), and was in accordance with a protocol approved by Johns Hopkins ACUC. Briefly, the mice were dark adapted for 12 hours before the ERG examination. Prior to the examination, mice were anesthetized under red light by intraperitoneal injection of ketamine and xylazine. Additionally, Proparacaine hydrochloride ophthalmic solution (0.5%) was used to anesthetize the cornea while tropicamide (1%) and phenylephrine hydrochloride (2.5%) solutions were used to dilate the pupil. Mice were placed on a temperature regulated heating pad throughout the recording session. ERG responses were evoked with a three-step protocol for a scotopic response (0, +5, +10dB light flashes) and four-step protocol for a photopic response (0, +5, +10 and 14dB light flashes) and recorded with an acrylic and gold metal contact electrode (LKC Technologies, USA). Mice were adapted for 10 min to a background of white light at an intensity of 30 cd/m^2^ and then photopic ERGs were performed. The ERG Data were analyzed with EMWIN 9.3.1 software (LKC Technologies).

### Mouse histological analysis

The mice were sacrificed after ERG data acquisition and immediately each eye was washed with 1×PBS (Life Technologies, USA) and formalin (Sigma-Aldrich, USA) respectively. The eyes were fixed for 48 hours in formalin and processed for histological analysis. The tissue was embedded in paraffin wax, and three μm sections were prepared using a Microtome. The sections were deparaffinized, hydrated, and finally stained with Hematoxylin and Eosin (H&E). Five fields per section were selected for examination using an Olympus BX-51 microscope (Olympus, USA) while three sections (30 μm apart) from each eye (n = 3 mice per group) were used for analysis.

### Immunofluorescence assessment of mouse retinal cones by cone arrestin staining

Wax embedded tissue sections were kept at 37 °C overnight. After deparaffinization, hydration and antigen retrieval, the sections were blocked with 5% BSA for 1 hour at room temperature. Sections were incubated with cone arrestin (1:1000; Millipore, Germany) primary antibody reconstituted 1 × PBS containing 1% BSA, overnight at 4°C in humid conditions. Subsequent to washings with 1 x PBS, the sections were incubated with corresponding FITC conjugated secondary antibody for 2 hours at room temperature, followed by three washings with 1 x PBS and finally staining with DAPI (Sigma-Aldrich, USA) for 15 min. Five fields per section were selected for examination using an Olympus lX-81 microscope (Olympus, USA) while three sections (30 μm apart) from each eye (n = 3 mice per group) were used for analysis.

### Web resources

1000 Genomes: http://www.internationalgenome.org/

ExAC: http://exac.broadinstitute.org/

gnomAD: http://gnomad.broadinstitute.org/about

Marshfield Data Base: https://www.biostat.wisc.edu/~kbroman/publications/mfdmaps/

OMIM: http://www.omim.org/

Retnet: https://sph.uth.edu/retnet/

## Supporting information

S1 Fig *CLCC1* region, pedigree, and haplotypes. Haplotypes of the *CLCC1* region of families 1–5 showing the *CLCC1* c.75C>A mutation, the *C1orf194* sequence variant, and surrounding microsatellite markers included in S2 Table. Cosegregation of the *CLCC1* c.75C>A mutation is shown for families 6–8.
(TIF)

S2 Fig SNP haplotypes across the *CLCC1* region. A. SNP Haplotypes of the 8 families extending across the region show conservation in a 322kb region of chromosome 1 are shown in yellow. B. schematic diagram of the conserved region showing the included genes.
(TIF)

S1 Table Two- point LOD scores of fine mapping markers. Autosomal recessive retinitis pigmentosa was analyzed as a fully penetrant trait with an affected allele frequency of 0.00001 and marker allele.
(XLSX)

S2 Table Intragenic CLCC1 haplotypes. SNP data for markers encompassing the CLCC1 gene indicate that a common founder mutation is responsible for the disease in each family.
(XLSX)

S3 Table Transcription factor binding sites altered by the 109492985G>T sequence variant. (XLSX)

S4 Table Summary of all variations in CLCC1 autozygous region identified in affected individuals. Allele frequencies of all other identified sequence variants identified excludes them as candidate causes of the disease.
(XLSX)
