# Supplementary figures and images for: Mutation in the intracellular chloride channel CLCC1 associated with autosomal recessive retinitis pigmentosa
Source: PLoS Genet. 2018 Aug 29;14(8):e1007504. doi: 10.1371/journal.pgen.1007504 (PMC6133373; doi:10.1371/journal.pgen.1007504)

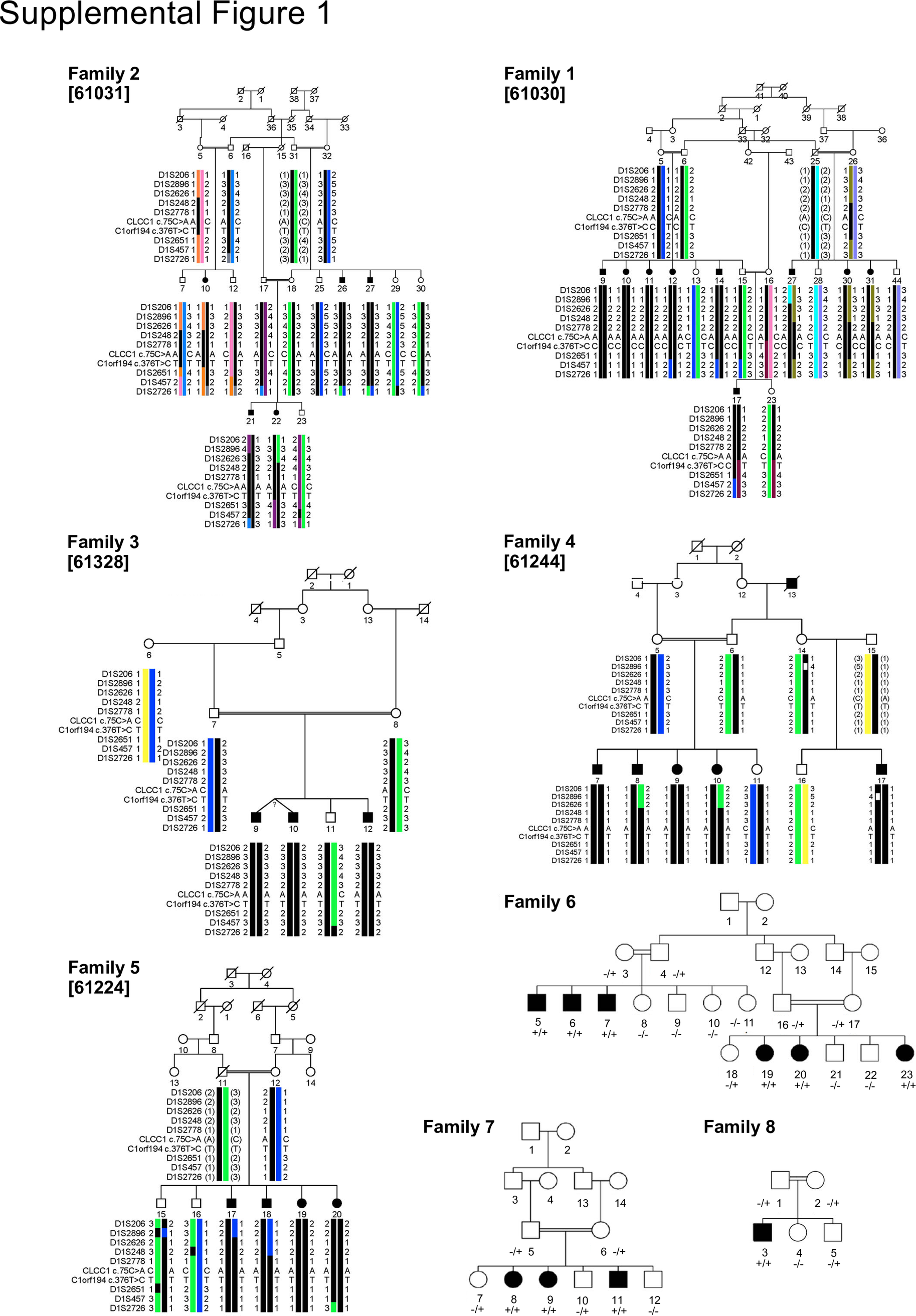

Supplement: S1 Fig — Haplotypes of the CLCC1 region of families 1–5 showing the CLCC1 c.75C>A mutation, the C1orf194 sequence variant, and surrounding microsatellite markers included in S2 Table. Cosegregation of the CLCC1 c.75C>A mutation is shown for families 6–8. (TIF) [file pgen.1007504.s001.tif]

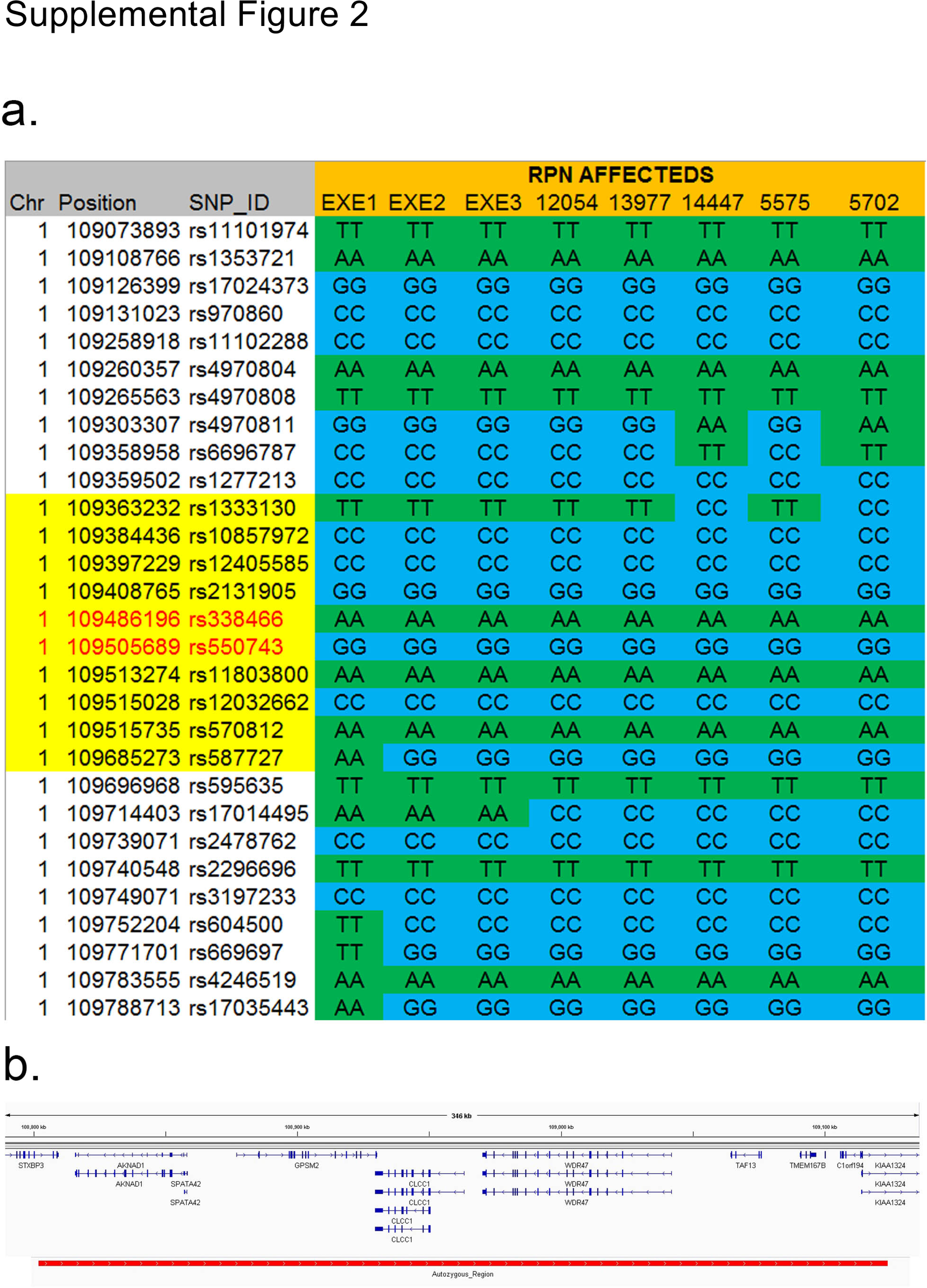

Supplement: S2 Fig — A. SNP Haplotypes of the 8 families extending across the region show conservation in a 322kb region of chromosome 1 are shown in yellow. B. schematic diagram of the conserved region showing the included genes. (TIF) [file pgen.1007504.s002.tif]
